# Production of Irregularly Shaped True-To-Life Microplastics with Embedded Optical Labels and Exemplary Application in an Ex Vivo Model

**DOI:** 10.1021/acs.est.5c08586

**Published:** 2025-08-28

**Authors:** Alissa J. Wieberneit, Sophia J. Baumann, Hannah Triebel, Sarah Dietrich, Nongnoot Wongkaew, Hayo Castrop, Antje J. Baeumner

**Affiliations:** † Institute of Analytical Chemistry, Chemo- and Biosensors, University of Regensburg, Universitaetsstrasse 31, Regensburg 93053, Germany; ‡ Institute of Physiology, 9147 University of Regensburg, Universitaetsstrasse 31, Regensburg 93053 , Germany

**Keywords:** microfiber, upconversion nanoparticles, irregular microplastic generation, fluorescence imaging, ex vivo kidney model

## Abstract

Ubiquitous in the environment, microplastics (MPs) are also taken up by all organisms. Possible implications are increasingly being studied, yet research is often limited by the use of idealized, spherical MPs. To better mimic MPs found in the environment, we demonstrate electrospun microfibers (MFs) as a possible precursor material, allowing for direct embedding of labels and simplified production of irregular microplastic (MP) fragments and fibers. Specifically, using polystyrene as a model polymer, MFs are doped with either organic (9,10-diphenylanthracene, DPA) or inorganic (upconversion nanoparticles, UCNPs) luminophores. Those optical labels allow for imaging under UV/vis or NIR excitation, respectively. Stable embedding is proven with minimal leaching over 35 days (DPA: 0.0023 wt %, UCNPs: 0.2 wt %). Mechanical disruption yielded MP fragments of (4 ± 3) μm in diameter for ball milling and fibers of (20 ± 20) μm in length for shear force exfoliation. While fibrous MPs were still too long for biological studies, the milled MPs are successfully applied ex vivo in mouse kidneys and are readily imaged in the tissue. Future studies on the biological impact will benefit from this approach, which offers a standardized method to produce traceable MPs that better resemble environmentally occurring MPs.

## Graphical abstract

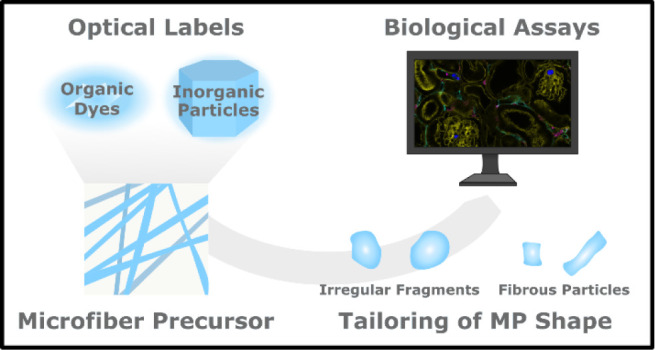

## 1 Introduction

Plastics are chemically inert and persist in the environment for prolonged periods. The annual rise in plastic production serves as an indicator of the increasing plastic consumption by industry and consumers.^1^ The degradation of plastics into particles in the environment involves mechanical and radiation-mediated breakdown, which eventually leads to the formation of microplastics (MPs) (<5 mm) and nanoplastics (<100 nm).^2^ The majority of MPs in aquatic or terrestrial ecosystems^3,4^ and in the human body consist of fragments and fibers,^5,6^ and has been detected in almost all human tissues.^7^ Most of the particles found are in the size range between 1 and 100 μm.^5^ In contrast to MPs found in the environment, most experimental studies are conducted with spherical polystyrene (PS) particles owing to their commercial availability.^4,8–10^ However, shape factors and not only material but also size must be included in studies as it has been demonstrated that the MP shape is a relevant factor when considering their effect on organisms.^4^ Therefore, studies should also aim to use true-to-life MPs, i.e., fragments and fibers.

In this sense, researchers face two challenges: distinct detection and production of MP fragments or fibers as tracer materials. For MPs found in the environment, the most common methods are based on microscopic evaluations coupled with spectroscopic methods for chemical analysis such as Raman or FT-IR.^11–13^ Additionally, attempts have been made toward a detection by subsequent fluorescence staining of environmental MPs.^12,14^ When used as tracer materials, current labeling strategies of MPs include carbon dots,^15^ inorganic particles,^16–18^ radioactive labels,^19^ and organic fluorophores as the most commonly used optical labels due to their high quantum yield^17,20^ and their cost-effectiveness. Drawbacks include their photobleaching, which complicates sample handling and long-term measurements.^21^ Moreover, the broad absorption and emission spectra of fluorophores can limit their suitability as labels in MP studies, as they may overlap with commonly used fluorophores in biological assays. To avoid the issue of photobleaching, upconversion nanoparticles (UCNPs) are often used as alternative optical labels due to their high photostability even at high power excitation for several hours.^22,23^ First proof-of-principles of UCNP-doped MPs have already been reported.^18^ Their low quantum yields of max. 13%^24^ are often compensated for by their excitation in the near-infrared (NIR), as the penetration depth of the excitation light is increased compared to UV/vis excitation by a factor of at least five, depending on the tissue studied.^25–27^ For the integration of labels into MPs, current strategies employ retroactive MP staining with dyes dissolved in solvent for a better integration of the label.^28,29^ Other options consist of simultaneous melting or dissolution of the polymer and dye.^30,31^ Addressing the challenge of the production of true-to-life MPs, researchers explore techniques such as lab-based weathering^9^ and sonication,^32^ resulting in particles of 1–3 μm and 0.1–1000 μm, respectively. Furthermore, ball milling (BM), often combined with liquid nitrogen cooling, is used to produce irregular particles,^6,9^ resulting in particles ranging from approximately 5 to 200 μm in size.^8,32–34^ First attempts toward the production of fibrous MPs have been made by sectioning commercial fibers with a cryotome, resulting in dimensions of 10 × 10 × 40 μm.^28^

Even though the discussed initial efforts have been made to produce true-to-life MPs, most biological studies still rely on the commercially available spherical particles. Here, recent studies using PS spheres have demonstrated a high correlation between the MP particle size and their accumulation in tissues, with sizes between 4 and 20 μm having a higher propensity.^35,36^ Accordingly, Stock et al. provided evidence for an increased uptake of 4 μm PS spheres in intestinal epithelial Caco-2 cells compared to those with a diameter of 1 μm, suggesting that the 4 μm particles can be taken up by phagocytosis and pinocytosis.^36^ Given its limited regenerative potential,^37^ the kidney is of particular interest in toxicity studies. It is known from transfusion studies that human and rat erythrocytes (7.5 μm in diameter), which slightly exceed the size of mouse erythrocytes (5 μm in diameter),^38^ are retained in the glomerular capillaries of mice and, subsequently, impede capillary blood flow. Consequently, MP particles around 5 μm in diameter may represent the upper size limit in terms of free glomerular capillary passage. In addition, erythrocytes are deformed and compressed during capillary passage,^39^ whereas the rigidity of MP particles presumably increases the risk of capillary blockade.^40^ Furthermore, irregularly shaped and fiber-like MP particles may be trapped at branching and junction sites of the glomerular capillary network.^41^ As the endothelium of glomerular capillaries is characterized by a dense glycocalyx,^42^ sharp and pointed MP particles may also penetrate the endothelial glycocalyx and, subsequently, be retained within glomerular capillaries. Accordingly, the mouse isolated perfused kidney model (MIPK) offers a good opportunity to investigate the passage of MPs through small blood vessels since it comes close to *in vivo* experiments and allows for a defined introduction of MPs into an intact kidney without being dependent on intestinal absorption.

This work aims to develop a new strategy for the generation of irregularly shaped true-to-life MPs based on electrospun microfibers (MFs), which are ideally suited for *ex vivo* and *in vivo* studies, as an alternative to commercially available uniformly spherical particles. Here, the MF precursors doped with either fluorescence dye or UCNPs are generated *via* electrospinning prior to being shortened into MPs using either a ball mill or an Ultraturrax. The fragmentation process is optimized to obtain MPs in a relevant size range (4–20 μm). The study focuses especially on the methods of fabrication and luminescent doping tailor-made for long-term bioimaging. The material properties are characterized in terms of size, shape, and luminescence properties using scanning electron microscopy (SEM), transmission electron microscopy (TEM), fluorescence and extinction spectroscopy, luminescence measurements, and micrographs obtained using confocal microscopy equipped with a two-photon laser. The MPs with the smallest size and narrowest size distribution are chosen for proof-of-principle studies carried out using the MIPK as an *ex vivo* biological model.

## 2 Materials and Methods

The list of chemicals is included in the Supporting Information.

### 2.1 Upconversion Nanoparticles as Optical Labels

#### 2.1.1 Synthesis of Core and Core–Shell Upconversion Nanoparticles

To obtain (Yb, Tm)-doped hexagonal β-NaYF_4_ core–shell UCNPs, both, the core particles and the shell precursor, were prepared according to a modified protocol by Schroter et al.^43^ Detailed instructions for a 5 mmol batch size of core particles and a 10 mmol batch size of shell precursor particles (regarding the lanthanide content) can be found in the Supporting Information.

For a shell thickness of approximately 3 nm, precursor solution (1.7 mmol) was added subsequently to the core particles (1 mmol) above 300 °C. The first addition (1 mL) was carried out at around 230 °C. At 315 °C, every 8 min, increasing amounts of the precursor solution (1 mL to 5 mL) were added to the core particles to ensure a uniform shell growth. After the final addition, the reaction was maintained at 315 °C for additional 8 min, followed by rapid cooling to room temperature. Further details regarding preparation and purification are provided in the Supporting Information. After purification, the oleate-coated UCNPs were stored in cyclohexane at 8 °C until further use.

#### 2.1.2 Surface Modification of Upconversion Nanoparticles for Dispersion in DMF:THF

For the embedding of the UCNPs into the MFs, the oleate-coated particles had to be stabilized in a mixture of 1:1 (v/v) DMF:THF in the spinning solution, containing PS. For this, a ligand removal reaction was carried out following a well-established protocol with slight adjustments.^44,45^ Here, DMF was added to the UCNPs dispersed in cyclohexane under constant stirring. The mixture was heated to elevated temperatures, and NOBF_4_ was added. After 30 min, the ligand removal reaction was completed, and the UCNPs stabilized in DMF were purified. For electrospinning, the resulting particle pellet was redispersed in a 1:1 DMF:THF mixture. Further details of the protocol can be found in the Supporting Information.

### 2.2 Production of Microfibers

PS MFs were produced by electrospinning. For this purpose, a spinning solution of 15 wt % PS (1050 mg, MW ≈ 280,000) in a 1:1 ratio of DMF and THF was stirred overnight in the dark. Unless stated otherwise, 0.3 wt % 9,10-diphenylanthracene (DPA, relative to the PS mass) or UCNPs in DMF:THF (mass concentration β_UCNP_ = 25 mg·mL^–1^, 17 wt % relative to the PS mass) were added to the spinning solution to obtain luminescent MFs. The MFs were electrospun with a rotary drum system (Linari NanoTech) under optimized conditions (flow rate: 30 μL·min^–1^ through an in-house-built 4-needle holder; needles: 18 G; distance to collector: 15 cm; voltage: 11–12 kV; ambient conditions: 22 °C; relative humidity below 35%; collection time: 3 h on aluminum foil). The MFs were stored in the dark under ambient conditions until use.

### 2.3 Production of Microplastic

#### 2.3.1 Ball Milling

MFs were milled using a planetary ball mill (PM100, Retsch). To ensure reproducibility of the process, all process parameters must be kept constant, *e.g.*, temperature, milling times, and amount of starting material. The MFs were filled into a 50 mL grinding jar together with 7 grinding balls (10 mm in diameter). Due to the high volume of the MFs, several pregrinding steps were necessary for 30 s at 550 rpm to break the fibers into fragments. After every 30 s step, more MFs were added to the grinding jar and broken down. When the jar was filled to around 1/3 with MF fragments, the main grinding was started. This process consisted of 9 cycles of 2 mins of milling at 550 rpm, each followed by 1 min of cooling on ice to prevent exceeding the glass transition temperature of PS. As an alternative to the use of 7 large balls, about 3000 balls of smaller balls (2 mm in diameter) were used to increase the frictional force at the expense of a lower impact force. The premilling and main milling steps remained the same. In a third setup, both milling processes were combined (pregrinding with bigger balls, 9 cycles with 9 larger balls, followed by 9 cycles with smaller balls).

#### 2.3.2 Cryo Milling

As an additional approach, MFs were broken down using cryogenic grinding (CryoMill, Retsch). Specifically, 400 mg of MFs were filled into a 50 mL grinding jar together with 8 grinding balls of 12 mm diameter. The material was precooled for 13 min with liquid nitrogen surrounding the grinding jar. The milling process consisted of 9 cycles (2 min each) of milling at 30 Hz plus 1 min of intermittent cooling per cycle.

#### 2.3.3 Ultraturrax

To produce more fibrous fragments, 20 mL of a 1 wt % soy lecithin solution was added to 240 mg of MFs. Shear force exfoliation was conducted using an Ultraturrax instrument (IKA T18 basic). For temperature control, the solution was kept on ice and shredded in 1 min cycles. Subsequently, the solution was cooled for 5 min between the shredding cycles (total shredding time: 7 min). Part of the resulting solution was filtered through a 10 μm metal mesh (Puri Select).

Unless stated otherwise, all experiments involving MPs were conducted using samples produced *via* the optimized ball-milling procedure. Prior to use, the MPs were emulsified in a 1 wt % soy lecithin solution by ultrasonication and vortexing.

### 2.4 *Ex Vivo* Application of Microplastic

#### 2.4.1 Mouse-Isolated Perfused Kidney

The MIPK model was used to evaluate the detectability of the produced MPs in the tissue samples. For MIPK experiments, the kidney was perfused *via* the renal artery with a buffer containing the plastic material of interest. The MIPK was performed according to Schweda et al.^46^ with PAX8/TetO-Cre/Ren1-flox (C57BL/6 background) mice as kidney donors and 0.5 mg of PS-DPA MPs added to the perfusion cycle. Specifically, the mice were sacrificed by cervical dislocation prior to the opening of the abdominal cavity. The aorta was clamped distal to the renal artery; the mesenteric artery was ligated, and a metal perfusion cannula was inserted into the abdominal aorta. Following the removal of the aortic clamp, the cannula was advanced to the origin of the right renal artery and secured in place. After ligation of the aorta proximal to the right renal artery, perfusion was started. The right kidney was excised, placed in a thermostated moistering chamber, and perfused at a constant pressure of 100 mmHg using a modified Krebs-Henseleit solution including physiological amino acids and glucose. The perfusion medium was continuously dialyzed against a larger volume of the same solution to maintain functional preservation. Finally, the kidney was perfused with 3% paraformaldehyde (PFA, pH 7.4) for further histological analysis.

#### 2.4.2 Immunohistochemistry

The PFA-fixated kidneys were soaked in cryo buffer (200 mL phosphate-buffered saline (PBS), 133 mL of 4% PFA in PBS, 32 g of saccharose) for 24 h before storage in liquid nitrogen. For fluorescence immunohistology, the kidneys were rapidly thawed at 37 °C and embedded in 3% agarose in PBS before cutting tissue sections of 150 μm using a vibratome (VT1200 S, Leica). After several washing steps with PBS, sections were blocked with 1% bovine serum albumin (BSA) in PBS including 10% horse serum prior to incubation with primary antibodies (CD31 (goat polyclonal, R&D Systems) and F4/80 (rat monoclonal, Abcam)) overnight at 4 °C. After washing with PBS, the secondary antibodies (Alexa Fluor 647 donkey antirabbit, Alexa Fluor 555 donkey antirat; Thermo Fisher) were added and incubated for 3 h at room temperature, followed by several washing steps with PBS. Following another blocking step with 0.5% Triton X in PBS for 1 h, a labeled phalloidin antibody (Phalloidin-iFluor 488 Reagent, Abcam) was incubated on the tissue sections for 2 h. Afterward, the sections were again washed several times using PBS. After immunohistochemical staining, the sections were mounted between two coverslips using a spacer (0.10 mm iSpacer, Sunjin Lab) for imaging. Kidney sections were analyzed using a confocal laser scanning microscope (LSM 710, Zeiss).

### 2.5 Characterization and Evaluation Techniques

#### 2.5.1 General Material Characterization

To determine the size distribution of the UCNPs, TEM analysis was performed on a 120 kV Philips CM12 microscope (FEI GmbH). For sample preparation, the particle dispersions (1 mg·mL^–1^) were dropped onto carbon-coated copper grids (400 mesh). MFs were spun directly onto the grid for TEM evaluation. To evaluate the size distribution, several micrographs were taken and analyzed using the software ImageJ (Fiji, v1.54f) with the plugin ParticleSizer by Thorsten Wagner, and Origin (Version 2022b). Size distribution analysis of MFs (doped and undoped) and MPs was performed on a 5–50 kV Zeiss/LEO 1530 SEM instrument (Zeiss) at an operating voltage of 5 kV. MFs or dry MP powder were directly placed on the SEM holder, while MP in 0.1 wt % soy lecithin solution was dropped onto the SEM holder and dried prior use. Afterward, the samples were sputtered with a gold-palladium mixture. Several SEM micrographs were taken at different spots; size evaluation was done manually using ImageJ (Fiji, v1.54f) and Origin (Version 2022b). Further details on the image evaluation process for TEM and SEM analysis can be found in the Supporting Information.

The determination of the UCNP mass concentration and composition was performed using inductively coupled plasma optical emission spectroscopy (ICP-OES) from SPECTRO (SPECTROBLUE FMX36). Calibration was carried out by using a multielement standard from PerkinElmer. The nanoparticle dispersion (10 μL) was dried and dissolved in conc. H_2_SO_4_ (0.5 mL). Then, HNO_3_ (1.5 M, 9.5 mL) was added. Lanthanide concentrations were calculated as the mean values from three subsequent measurements. Colloidal stability and surface potential of particle solutions were analyzed using the Zetasizer Nano ZS (Malvern Panalytical) to measure dynamic light scattering (DLS) and zeta potential. All samples were measured three times. Zeta potential measurements of MP samples were carried out in 10 mM KNO_3_ (β_MP_ = 0.5 mg·mL^–1^). Luminescence measurements of UCNP-containing samples were performed using a home-built setup, equipped with a 980 nm laser module (200 mW, 150 W·cm^2^, continuous wave (cw)) from Picotronic, and a spectrometer for UV/vis from Broadcom (*Q*_mini_). Spectra were collected from 225 to 1000 nm, with a short-pass filter (cutoff 850 nm) and a bandpass excitation filter (cutoff 900 nm) to record upconversion luminescence, using the software Waves (RGB Photonics). Luminescence measurements of UCNPs dispersed in cyclohexane (β = 10 mg·mL^–1^), PS MP, PS MP with the addition of UCNPs, PS-UCNP MP, and UCNPs in both double-distilled water and 0.1 wt % soy lecithin were conducted. If not stated otherwise, the MP samples were stabilized in a 1 wt % soy lecithin solution with a mass concentration of 4 mg·mL^–1^ and further diluted in a 1:10 ratio with double-distilled water. UCNPs dispersed in DMF were added to the aqueous solutions to reach a concentration of approximately 50 μg·mL^–1^.

Fluorescence measurements were performed on an FS5 spectrophotometer (Edinburgh Instruments). Unless stated otherwise, the excitation wavelength was fixed at 405 nm, and the emission was recorded between 420 and 600 nm in 1 nm steps. The bandwidths for excitation and emission were set to 2 nm.

Fourier transform infrared spectroscopy (FT-IR) of knife-coated polymer foils, electrospun MFs, and MPs was performed with a Cary 630 FTIR from Agilent using dry materials.

Photographs of the MF-mat were taken with a DLSM camera (Canon EOS R6) and a macrolens equipped with a NIR filter to block the laser.

#### 2.5.2 Evaluation of Optimal Fluorophore Concentration

Knife-coated polymer foils were used for a simple analysis of the fluorophore behavior in the PS matrix and the optimization of the doping concentration. Based on the protocol optimized for electrospinning, polymer solutions containing 15 wt % PS (MW ≈ 280,000) in a 1:1 ratio of DMF:THF with various fluorophore concentrations (0.01, 0.05, 0.1, 0.2, 0.3, 0.5, 0.6, 0.8, 1 wt %) were stirred overnight in the dark. To determine the influence of solvents on the fluorescence spectra, solutions with either a 1:1 mixture of DMF:THF or pure chloroform were prepared while keeping the polymer and dye concentrations constant. The polymer foils with an approximate size of 5.5 × 11.0 cm were produced with an in-house-built knife coater. The thickness of the coatings was set to 30 μm with the help of spacers. The polymer films were dried for 3–4 h at 80 °C in an oven before use. To measure the absorbance and fluorescence, the foils were cut with a laser cutter (detailed information can be found in the Supporting Information) and glued with double-sided adhesive tape (Tesa) to the backside of a bottom-less black 96-well plate (Greiner Bio-One). Afterward, absorbance spectra (300–700 nm, step width 1 nm) and fluorescence spectra (Table S1) were measured with a BioTek reader (Agilent, USA). The mean value and the standard deviation of the data from 8 wells were calculated and plotted against the doping concentration.

To investigate the photostability, the knife-coated PS-DPA foils were excited with a xenon lamp (λ_ex_ = 385 nm, λ_em_ = 410 nm, slit size of 16 nm) for 6 h. The resulting fluorescent signal was measured every 5 s with an AMINCO Bowman Series 2 spectrofluorometer (AB2) with fiber optics (Thermo Spectronics, now Thermo Fisher Scientific). The initial fluorescent signal was normalized to 100%.

#### 2.5.3 Integrity of Luminescent Labels

PS-DPA MFs (515.82 mg) or PS-UCNP MFs (325.1 mg) were stuffed in a dialysis tube (cutoff: 12–14 kDa, Spectrum Laboratories Spectra/Por) and transferred to a Schott flask (100 mL). Double-distilled water (80 mL) was added, and the dialysate was exchanged at days 1, 2, 7, 10, 14, 28, and 35. Afterward, the dialysate was completely evaporated, and the flasks were either rinsed with 1.5 mL of chloroform for PS-DPA MFs, or conc. H_2_SO_4_ (0.5 mL), followed by the addition of HNO_3_ (1.5 M, 9.5 mL) to prepare the samples for ICP-OES measurements in the case of PS-UCNP MFs. For the fluorophores, fluorescence spectra were recorded as described above between 420 and 600 nm. Afterward, the percentage of leaching was determined using the integration of the signal between 421 and 441 nm. Based on a calibration curve between 5 × 10^5^ and 1 × 10^3^ wt %, the total mass of leached DPA was calculated. In the case of PS-UCNP MFs, the mass loss was determined by calculating the ratio between the mass determined with ICP-OES and the theoretical total mass of UCNPs inside the MFs (calculated from the mass used, assuming a homogeneous distribution of the UCNPs within the MFs).

For measurements under different environmental conditions, PS-DPA MP and PS-UCNP MP were suspended in a concentrated SDS solution (1 mg·mL^–1^) with a mass concentration of 4 mg·mL^–1^. The MP samples were diluted in a 1:10 ratio in double-distilled water, NaCl solution (150 and 500 mM), acidic conditions (1 M HCl), neutral conditions (pH 5.5, MES buffer), and basic conditions (pH 10, TE buffer). All samples were measured at RT and 37 °C. PS-DPA MP samples were measured with a BioTek reader (Agilent, USA, λ_ex_ = 405 nm, λ_em_ = 430–600 nm, step width 1 nm, *n* ≥ 3). The maximum fluorescence emission was averaged over the emission between 432 and 436 nm. PS-UCNP MP samples were measured with the setup described above for UCNP luminescence measurements (*n* = 3). The spectra were normalized to the 477 nm emission and further integrated in the area between 308 and 837 nm. For both, the luminescence was normalized to the signal determined in water at RT.

#### 2.5.4 Microscopic Evaluation of the Microplastic and Tissue Samples

The microscopic imaging and analysis of the doped MFs and their resulting MPs were performed using a confocal laser scanning microscope LSM710 (Zeiss) equipped with a Ti: Sapphire Chameleon Ultra Vision II laser (600–1200 nm; 4 W, 1 fs–cw, Coherent), an UV laser diode (405 nm, 52 mW, cw), a multiline argon laser (458 nm, 488 nm, 514 nm, 144 mW, cw), a DPSS laser (561 nm, 60 mW, cw), and a HeNe laser (633 nm, 9 mW, cw). The microscope was controlled by ZEN 2.3 SP1 software (Zeiss). Images were taken with 40× magnification. Imaging of UCNP-doped material was performed under 980 nm irradiation (1200 mW) in laser scanning mode (41.26 μs·pixel^–1^). The laser operated at 50% of the maximum power for all samples. The emitted light was collected with a short-pass filter for 485 nm (SP485 IR+). For visualization of the PS-DPA MFs and the resulting MPs, the UV laser diode was used. The laser excited the material at a wavelength of 405 nm using 1.00% laser power in the laser scanning mode (0.66 μs·pixel^–1^). The analysis of the tissue samples used all lasers for several excitation wavelengths (405, 488, 543, 647, and 980 nm) to display the structure of the tissues as well as the MPs. Images were processed with ZEN lite, ImageJ (Fiji, v1.5k), and CorelDRAW (Version 24.2.1.446).

## 3 Results and Discussion

For the production of luminescent, irregularly shaped MP, a suitable precursor material is required. Using a precursor which is already a micromaterial in a top-down approach is expected to enhance production efficiency.^47^ It is suggested that MFs are promising candidates as they have two dimensions in the microscale, are nonspherical, and can easily be mass-produced from many polymers also found as contamination in the environment, including PS, poly­(methyl methacrylate) (PMMA), polyethylene terephthalate (PET), or nylon.^3,12,48–50^ Furthermore, the challenges of postproduction doping can be avoided by directly embedding optical labels in the polymer solutions.^51^ To demonstrate such capability, PS as commonly used MP material was chosen as a model polymer.^4^ A range of optical labels was investigated, and the resulting MPs were fully characterized and finally applied to an *ex vivo* kidney model.

### 3.1 Application-Related Selection of Optical Labels

#### 3.1.1 Fluorescent Labeling with DPA

For the proper selection of a fluorophore dopant in MPs, key criteria include a high quantum yield and good photostability, solubility within the polymer solution, and an excitation wavelength that will avoid interference with fluorophores used in standard cell studies. The fluorophores perylene and DPA were chosen with quantum yields of minimum 0.82 and 0.95, respectively, high molar absorption coefficients at 405 nm (Table S2), and reported high photostability in polymer matrices.^20,52^

When absorbance and fluorescence spectra were compared (Figures 1A and S1), DPA proved to be more suitable for the requirements due to narrower excitation and emission peaks. Furthermore, DPA showed no emission under excitation at 488 nm in contrast to perylene (Figures S1C,D and S2). This would allow imaging of MPs in tissues with strong autofluorescence at 488 nm, such as the kidney, or the use of this separated wavelength for additional antibody staining, *e.g.*, phalloidin for actin structures, without spectral overlap.

**Figure 1:**
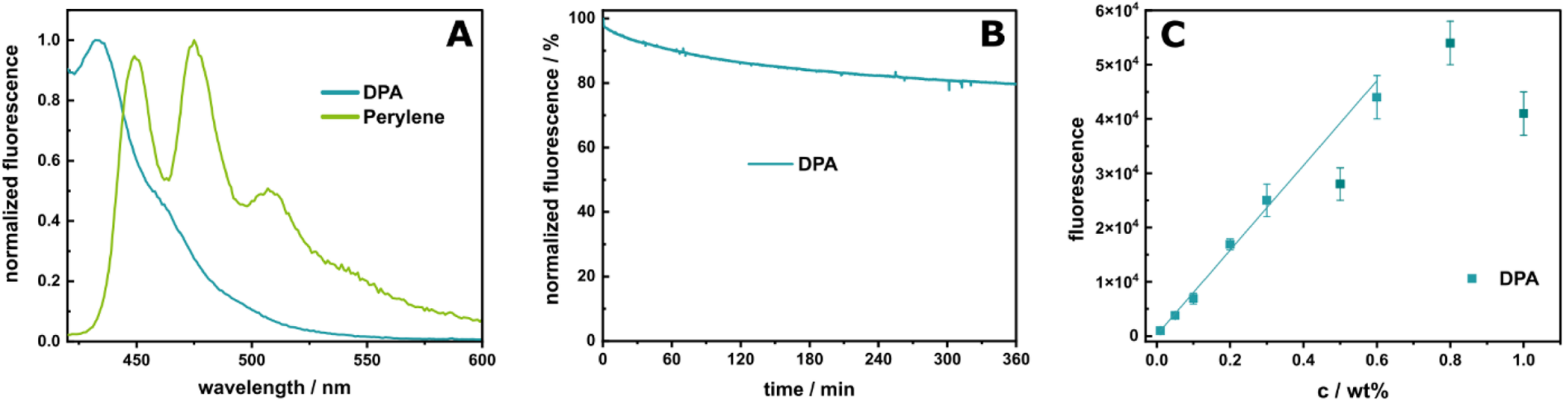
(A) Fluorescence spectra of MP doped with DPA (0.3 wt %) and perylene (1 wt %), dispersed in a 1 wt % soy lecithin solution at a concentration of 2 mg·mL^–1^ (1:10 dilution, λ_ex_: 405 nm). (B) Photobleaching study displaying the percentage decrease in the fluorescence of DPA over time. Conducted on knife-coated PS sheets with a doping concentration of 1 wt % DPA, λ_ex_: 385 nm, λ_em_: 410 nm. (C) Dependency of fluorescence signal on the doping concentration of DPA. The maximum fluorescence signal was plotted against the doping ratio, with a linear correlation of up to 0.6 wt % (*R* ^2^: 0.993, λ_ex_: 405 nm). Measurements were performed with knife-coated polymer sheets (*n* ≥ 20). A zoom-in to the lower concentrations is shown in Figure S3.

By exposing DPA embedded in a PS matrix to UV light from a Xenon flash lamp for 6 h (Figure 1B), which corresponds to roughly 60 h of direct sunlight,^53,54^ a loss of only about 20% in fluorescence emission was observed. Therefore, DPA can be considered reasonably photostable, and sample preparation and handling can occur under ambient light, significantly simplifying all procedures. In addition to photostability, the effectiveness of DPA as a fluorescent label also depends on the concentration. For an optimal doping concentration, various criteria need to be considered including the obtainable signal-to-noise ratios, the avoidance of leaching, and self-quenching.^20^ Therefore, the concentration dependence of DPA’s fluorescence signal was investigated (Figures 1C, S3 and S4), revealing a linear correlation up to 0.6 wt %. At higher concentrations, fluorescence quenching was observed. Hence, 0.3 wt % of DPA was chosen as the optimal labeling concentration, providing sufficient fluorescence and avoiding concentration quenching.

#### 3.1.2 Upconversion Nanoparticles

While fluorophores offer many advantages for labeling MPs, their broad absorption and emission bands, as well as their susceptibility to photobleaching, can hinder multiplexing and long-term imaging applications. In contrast, the inorganic UCNPs presented here provide another detection strategy, offering sharp, tunable emissions, exceptional photostability, and low autofluorescence due to their excitation in the NIR range.^21,22^ Hence, the feasibility of using them for MP labeling was investigated. Core–shell UCNPs with the composition NaYF_4_(25%Yb,0.3%Tm)@NaYF_4_ were successfully synthesized (Figure 2A). The shell with a mean thickness of 3 nm is optically inactive to enhance the optical emission by reducing energy migration to the surface and subsequent surface quenching (Figures S5 and S6).^55,56^ The UCNPs are uniform in shape and size with a diameter of 22 nm that can be reproducibly achieved (first batch: (22.3 ± 1.5) nm; second batch: (20.7 ± 1.3) nm). The uniformity and colloidal stability were confirmed by DLS measurements (Figure S5C,F,I), which did not change after the subsequent transfer of the particles into a DMF:THF mixture (Figure S5F,I). The solvodynamic diameter decreased due to the removal of the oleic acid ligand, yielding bare, ligand-free nanoparticles. Upon embedding into the PS MP, the upconversion luminescence spectrum was compared to the one in cyclohexane (Figures 2B, S6 and S7). The spectral attributes are identical, with an increase in the 800 nm emission. This, and the peak emerging at around 820 nm, can be attributed to scattering of the excitation and emission light by the MP particles, as confirmed by control experiments, showing the same peak in solutions with only PS MP and PS MP mixed with UCNPs (Figure S7A,B). Also, soy lecithin as a stabilizing solvent shows slight scattering effects, as shown in Figure S7C, when compared with UCNPs in double-distilled water. The abrupt cut at 900 nm is a consequence of the selected low-pass filter required to protect the detector from the excitation light (cutoff at 900 nm). Further details regarding the characterization of the nanoparticles can be found in the Supporting Information.

**Figure 2:**
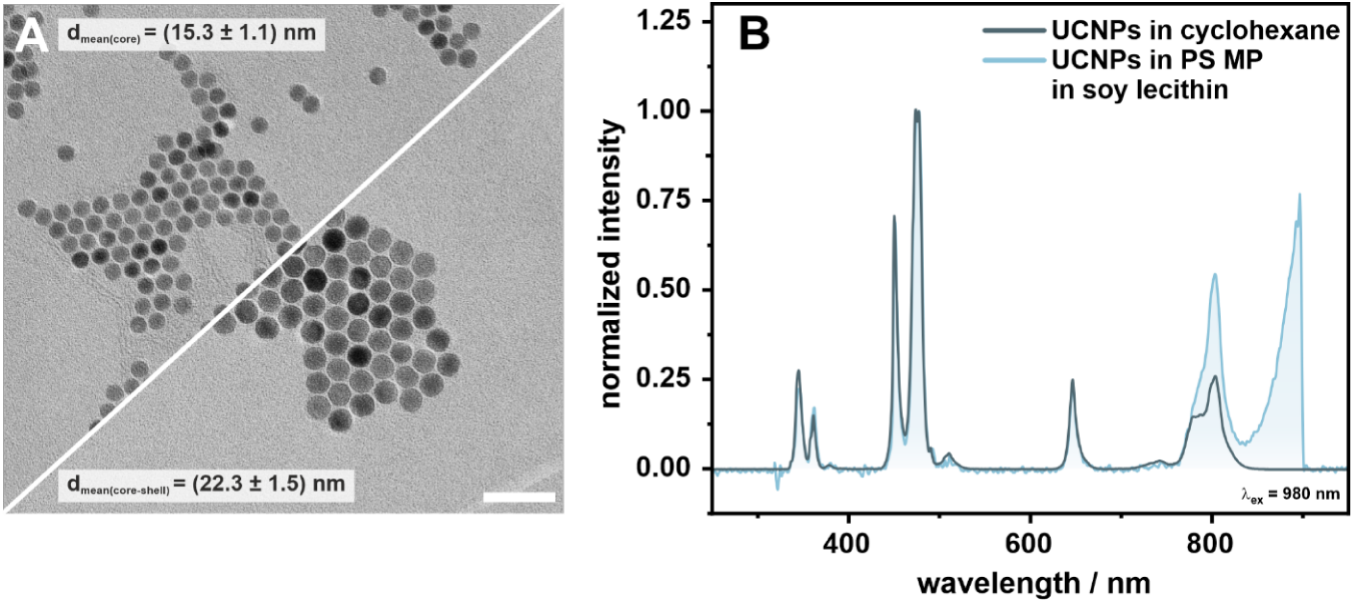
Characterization of UCNPs used as dopants in the PS matrix. (A) TEM micrographs of core (NaYF_4_:Yb,Tm) and core–shell (NaYF_4_:Yb,Tm@NaYF_4_, first batch) nanoparticles. TEM analysis results in a diameter of (15.3 ± 1.1) nm and (22.3 ± 1.5) nm, respectively, corresponding to a shell thickness of (3.5 ± 0.9) nm. Scale bar = 100 nm (B) Luminescence measurement of NaYF_4_:Yb,Tm@NaYF_4_ UCNPs in cyclohexane (dark blue, first batch) and in PS MP in 1 wt % soy lecithin (light blue, β_initial_ = 4 mg·mL^–1^, 1:10 dilution) normalized to the 477 nm emission (λ_ex_: 980 nm, 150 W·cm^–2^, cw).

The obtained UCNP-doped MFs could offer noninvasive imaging and elemental analysis as detection mechanisms for the resulting MP. In the latter case, ICP-OES or ICP-MS can be used for highly sensitive detection after digestion of the sample.^17,57^ With regard to noninvasive imaging, the UCNP-doped MPs support NIR excitation, hence avoiding autofluorescence of the tissue and interferences with standard fluorescent probe detection.^25,58,59^ Thereby, they also enable deep tissue imaging due to the increased penetration depth of the excitation light lying in the optical window of the tissue.^25,26^ The emission of Tm-doped UCNPs at 800 nm is also within this optical window, making this a suitable emission wavelength for detection.^24^ Furthermore, in contrast to organic dyes, the emission bands are very narrow (Figure 2B) and can be finely tuned by changing the lanthanide doping.^60^ Although they exhibit comparably low quantum yields to other luminescent probes,^24^ higher doping rates in the polymer matrices can overcome this drawback.^61^ Here, it must be noted that high doping concentrations of inorganic particles can alter the physicochemical properties of the MPs, resulting in differences in the uptake of the MPs.^17^ These effects can be minimized by the direct embedding of the UCNPs into the polymer matrix rather than surface-bound labeling approaches. The direct embedding was successfully performed in this study but might require further optimization.

### 3.2 Microfibers as Precursors for the Production of True-To-Life Microplastic

#### 3.2.1 Stable Embedding of Optical Labels in the Polymer Matrix

Following the successful identification of suitable optical labels, the subsequent step was the production of microfibers doped with DPA and UCNPs, as well as the assessment of the stability and integrity of the labels. The PS MFs obtained with the optimized spinning parameters for efficient production and bead-free fibers (summarized in Section 2) show a narrow size distribution in SEM analysis, where the thickness of the fibers is independent of the dopant (DPA: (0.95 ± 0.13) μm, UCNPs: (1.0 ± 0.2) μm; Figures 3A,B and S8A). They appear with a rough surface with occasional fissures, regardless of the dopant (Figure S9). In earlier research with thin polyvinylpyrrolidone MFs, UCNP doping distribution in MFs could be evaluated by TEM,^61^ but the significantly thicker PS fibers increased electron scattering and phase contrast, making a visualization of the particles within the fiber impossible (Figure S8B).^62^ Instead, SEM imaging at high magnification of fissures within the MFs (Figures 3D and S8A) could show spherical structures with a diameter that aligns with that of the UCNPs. Moreover, confocal laser scanning microscopy indicated a successful homogeneous distribution of both optical labels within the polymer matrix (Figure 3B,E), which can also be seen in a photograph of the PS-UCNP MF mat (Figure S8C).

**Figure 3:**
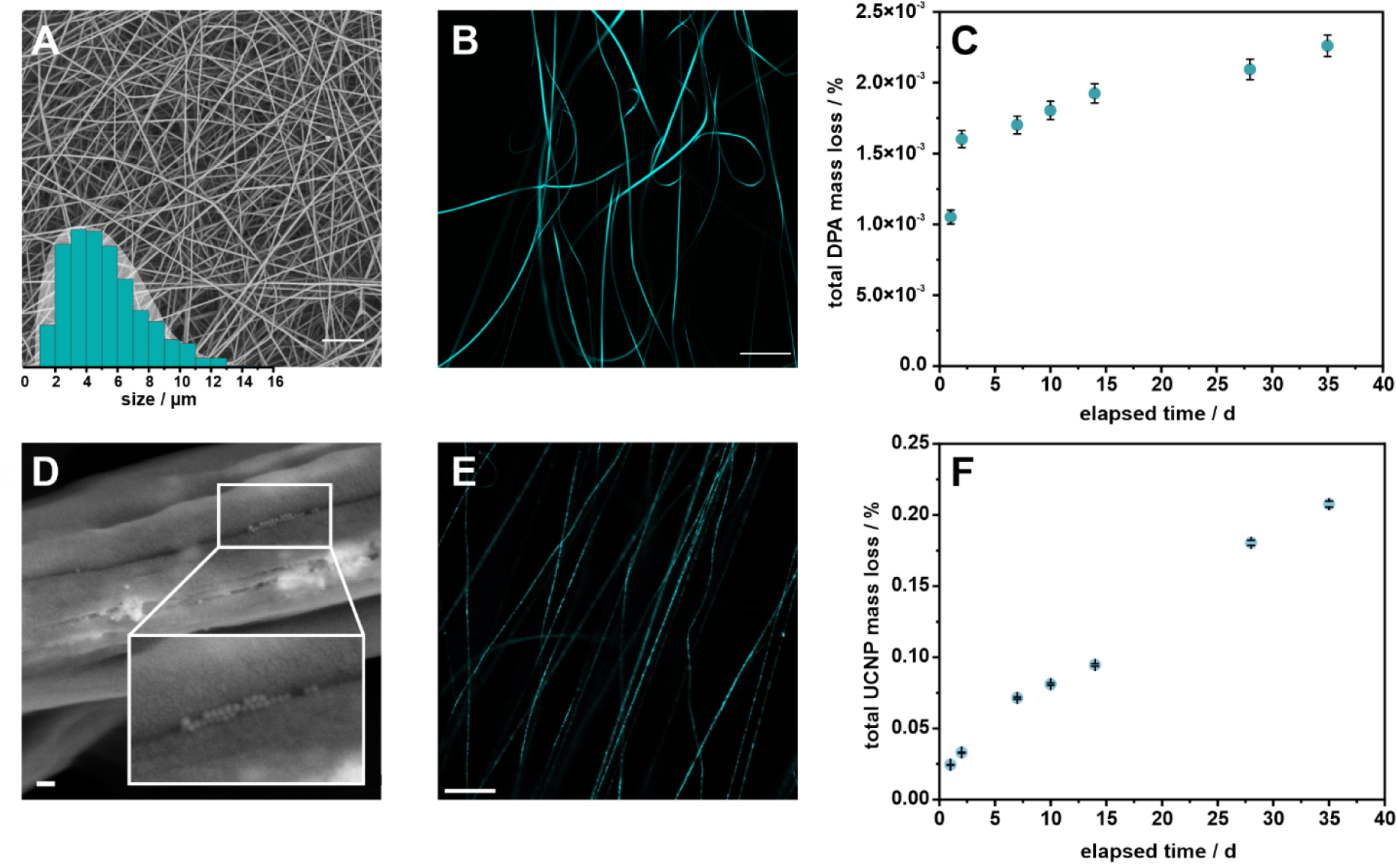
PS MFs doped with 2 wt % DPA (A–C) and 17 wt % UCNPs (D–F). (A) SEM micrograph of PS-DPA MFs with the corresponding size distribution; scale bar = 20 μm. (B) MFs excited under a fluorescent microscope; scale bar = 50 μm, λ_ex_: 405 nm, 52 mW, 1% laser power. (C) Leaching study investigating the total release of DPA from the MFs into aqueous solution over a period of 5 weeks, monitored by fluorescence measurements. (D) SEM micrograph of PS-UCNP MFs. One spot where UCNPs can be seen is zoomed out. Scale bar = 100 nm. (E) Microscopic image of the PS-UCNP MFs; scale bar = 50 μm, λ_ex_: 980 nm, 1200 mW, 50% laser power. (F) Leaching study of PS-UCNP MFs with total mass loss of UCNPs determined by ICP-OES.

To evaluate the stability and integrity of the optical labels within the PS matrix, a series of experiments was conducted. Fluorescence spectra of DPA were recorded at different stages during the production process, confirming the chemical integrity: dispersion in chloroform, in the spinning solution, incorporation in knife-coated PS polymer foils, and in the final MP (Figure S10). Due to strong scattering effects, however, spectral analysis of the MFs was not feasible. To investigate the effectiveness of the direct embedding approach in retaining the optical labels within the polymer matrix, a leaching study was performed. The MFs were soaked in water and monitored over a period of 5 weeks (Figure 3C,F). The results indicate minimal to possibly no leaching, with DPA and UCNPs showing mass losses of only 0.0023 and 0.2 wt %, respectively. Notably, PS-DPA-MFs and PS-UCNP-MFs exhibit different leaching behaviors. While DPA shows an initial increase in fluorescence followed by stagnation (only about 1 · 10^–3^% in 34 days), UCNPs show continuous but overall low leaching throughout the whole study. These differences are likely attributed to the hydrophobicity of DPA, whereas ligand-free UCNPs can be attributed as hydrophilic. However, the total mass loss of UCNPs is still negligible, supporting the conclusion that both labels are stably embedded in the polymer matrix and that a constant luminescent signal over long-term studies can be assured. Given that UCNPs contain fluoride ions, potential cytotoxicity was also considered. The detected concentration of UCNPs in solution was (0.20 ± 0.02) μg· mL^–1^, corresponding to approximately 4 μM of total fluoride released (in a total volume of 560 mL double-distilled water, following eq S1–S4). This concentration is 3 orders of magnitude below reported effect concentrations of fluoride and 2 orders of magnitude lower for UCNPs.^63–65^ Accordingly, UCNPs embedded into PS can be considered biologically and physiologically benign. Further optimization may include an initial washing step for DPA-MFs to remove loosely bound dye, whereas adjustments to the spinning parameters could enhance UCNP integration and further reduce leaching.

In addition to evaluating the long-term retention of the labels in the PS matrix, the functional stability of the embedded optical labels was examined under varying environmental and physiological conditions at RT and 37 °C (double-distilled water, 150 mM NaCl, 500 mM NaCl, 1 M HCl, pH 5.5, 10; Figure S11). As expected, the fluorescence signal of DPA can be quenched by chloride ions in the environment.^20^ However, even at high concentrations (500 mM NaCl, 1 M HCl), which are not to be expected under physiological conditions, a sufficient signal intensity of about 75% remains. In the two buffer solutions (pH 5.5 and pH 10), even an increase in signal intensity was observed. These fluctuations can easily be compensated for by adjusting the laser power during microscopic analysis. UCNPs, in contrast, exhibit a stable signal under all conditions, which further highlights their exceptional photostability. While a temperature dependency could be expected for both labels,^20,66^ only DPA at pH 10 showed a significant difference between RT and 37 °C, but with an increase in the signal. Taking these results into account, qualitative measurements can be performed under all of the investigated conditions.

#### 3.2.2 Production of Microplastic with Control of Particle Shape

Following the successful and stable integration of the optical labels, the next step focused on the production of MPs starting from MFs using typical top-down approaches such as ball milling, cryo milling, or alternatively shear force exfoliation with an Ultraturrax (UT). Particle shapes resulting from these production methods are expected to be highly irregular and possibly even fibrous.

The comparison of the top-down approaches revealed systematic differences (Figures 4 and S12) as only with shear force exfoliation could the fibrous shape be maintained. All particles obtained by milling showed an increased diameter in SEM analysis compared to the fibers as the polymers tend to be compressed during milling.^67^

**Figure 4:**
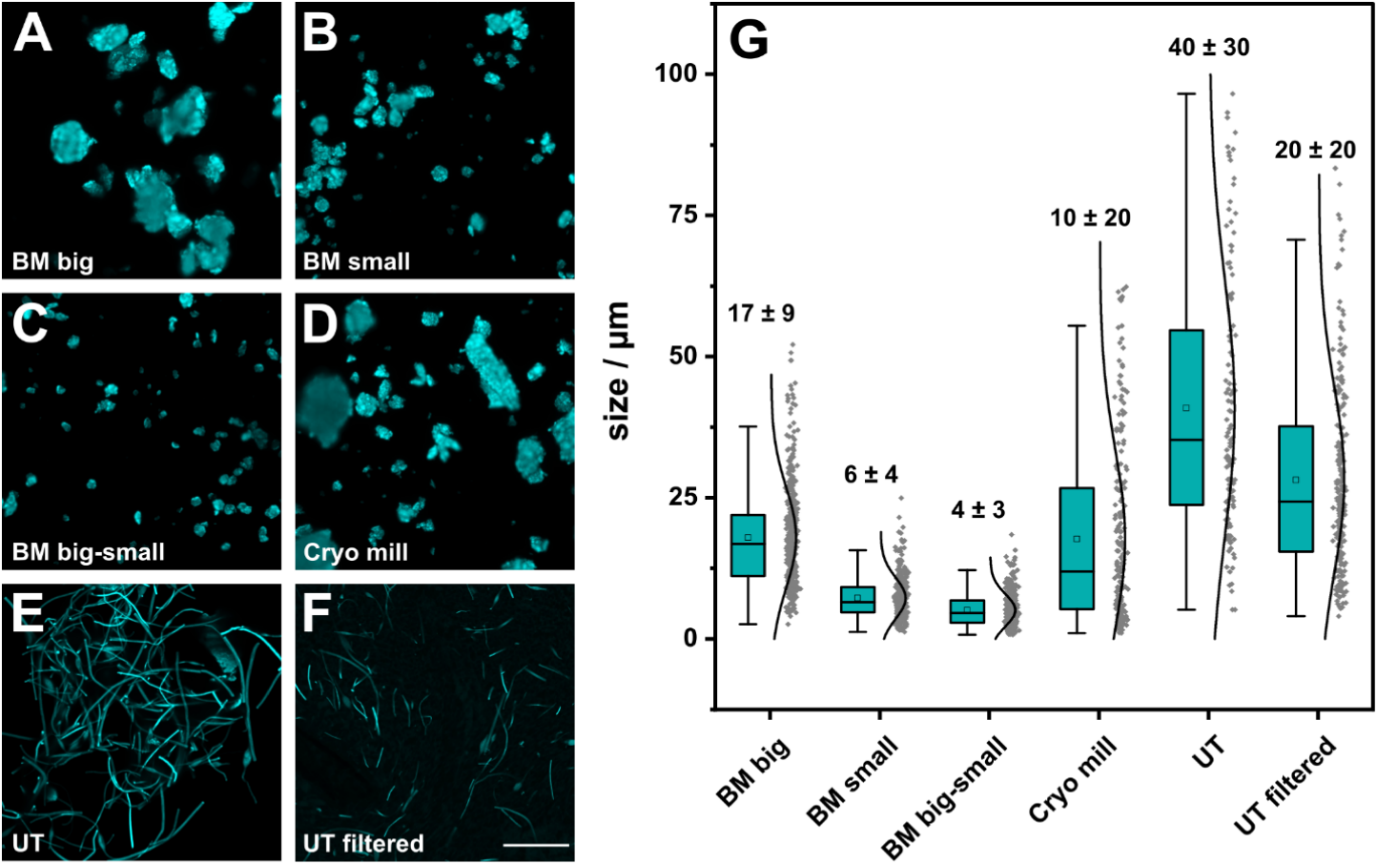
Comparison of light microscopy images of the different MP production approaches (scale bar = 20 μm, λ_ex_: 405 nm) and the resulting size distribution for PS-DPA MP. (A) MP generated by a planetary BM (50 mL grinding jar; 7× 10 mm grinding balls) after 9 milling steps of 2 min. (B) MP generated by a planetary BM (50 mL grinding jar, 3000× 2 mm grinding balls) after 9 milling steps of 2 min. (C) Particles from (A) after a second milling step with 9 cycles of 2 min each with small balls (50 mL grinding jar, about 3000× 2 mm grinding balls). (D) MP obtained from a cryo mill under liquid nitrogen cooling (50 mL grinding jar, 8× 12 mm grinding balls) after 9 milling cycles of 2 min grinding and 1 min intermediate cooling. (E) MP fibers produced by an UT in 1 wt % soy lecithin solution before and (F) after filtration through a 20 μm metal sieve. (G) Plot of the size distribution of the different production methods; *n* > 400.

The size of the balls affects the milling outcome (Figure 4) as also described elsewhere.^68,69^ Larger balls (diameter of 10 mm) grind the material down due to impact forces^70^ and hence lead to larger flakes with a broad size distribution of (17 ± 9) μm (Figures 4A,G and S12A). Smaller balls lead to a reduction in the size of (6 ± 4) μm (Figures 4B,G and S12B). To avoid longer cooling times between milling cycles required for heat dissipation in the case of the small BM process, a combination of both processes was tested. The sample was premilled with the large balls for particle formation and then grinded down with the small balls (Figures 4C,G and S12C). With this optimized ball milling procedure, the smallest particles were achieved, with a high reproducibility between different batches. For undoped PS MP, diameters of (6 ± 4) μm (Figure S13A) were achieved, while PS-DPA MP yielded particles with sizes of (4 ± 3) μm (Figure 4C,G) and (6 ± 3) μm (Figure S13B). For PS-UCNP, MPs of (6 ± 3) μm (Figure S13C) were produced. Those results are similar to other two-step procedures reported in the literature.^69^ The morphology of all PS MPs, PS-DPA MPs, and PS-UCNP MPs (Figures 4C,G and S13) is comparable, independent of the doping. Furthermore, the spherical structures observed in the PS-UCNP MFs (Figure 3D) could not be observed in the milled MP (Figure S13C). Additionally, the zeta potentials of PS MP, PS-DPA MP, and PS-UCNP MP are comparable, all carrying a similar negative surface charge (Table S3), therefore indicating a similar uptake behavior regardless of the dopant.

Cryo milling typically yields smaller particle sizes, can reduce unwanted side reactions, and makes the material more brittle, by keeping it below the glass transition temperature during the whole process^71,72^ and has already been tested for MP production.^8^ Unfortunately, in contrast to those findings, only large particles with a very broad size distribution were obtained here (Figures 4D,G and S12D). It is assumed that the fluffy nature and, hence, the low density of the MFs prevented effective cryo milling. As described in Section 2, refilling the grinding jar is necessary to achieve optimal milling results. However, ice crystal formation can occur during the refilling due to the lowered temperatures, which itself can also negatively impact the milling results. In future work, refining strategies under cryo conditions will be studied. Only after optimization, the potential of cryo-cooling on the MP size, shape characteristics, or increased homogeneity can be effectively evaluated and compared to the two-step BM process.

UT treatment of the MFs essentially leads to the cutting of fibers into shorter pieces (Figures 4E–G and S12E,F). Regarding the targeted biologically relevant size range of 4 – 20 μm for MPs,^35,36^ these fibers are too long with a length of (40 ± 30) μm, even after filtration with (20 ± 20) μm. Therefore, further optimization is necessary before those particles can be used in biological assessments. A reduction in size could be realized, for example, by extending the shredding time, lowering the temperatures, or using higher shear forces. These optimizations will be addressed in future work.

Chemical integrity of PS during the production procedure was evaluated by recording FT-IR spectra of PS polymer foils, MFs, and MP (Figure S14). Here, no changes between the different samples were observed, indicating the integrity of the polymer during the whole production process. Hence, the chosen fabrication parameters were effective in keeping the temperature within the vessels below the glass transition temperature, which is important to ensure the integrity of the embedded dopants as well.^73,74^

To highlight the relevance of producing irregularly shaped MPs, a visual comparison was conducted by using SEM between the MPs developed in this study and commercially available spherical MPs commonly used in MP research. The commercial MPs display a uniform size, smooth surface, and perfect spherical shape (Figure 5A), in strong contrast to the irregular morphology of the MPs produced here (Figures 5B,C and S12). This morphological distinction is critical, as particle shape influences both environmental transport behavior^75,76^ and biological interactions. Literature reports that rough-edged particles influence the uptake and potential damages to tissue and may increase inflammation, oxidative stress, or cell membrane damage.^77,78^ The MPs developed here closely resemble MPs isolated from the environment as described in the literature,^9,79,80^ even though no standardized definition or classification system is available for the discrimination of different particle shapes and classes.^13,35^ They thereby offer a relevant model for the investigation of shape-dependent biological effects,^81^ which will be explored in future studies.

**Figure 5:**
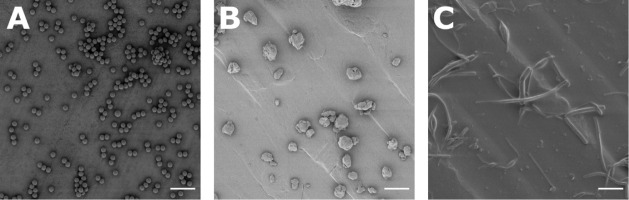
Shapes of MPs as determined by SEM. (A) Commercially available spherical MP particles were obtained from microParticles GmbH. (B) MP produced by BM from PS MFs doped with 0.3 wt % DPA. (C) Fibrous MP produced by an UT. Scale bar = 20 μm.

In order to validate the MFs as a reliable precursor for the production of irregular PS MPs, a comparative analysis with techniques described in the literature was conducted, discussing the benefits and drawbacks of each approach (Table S4). Besides lab-based weathering and sonication,^9,32^ the majority of reported techniques are based on BM. All reported particles exhibited a rough surface and heterogeneous size distribution. However, the resulting particle sizes varied considerably with diameters ranging from 1 to 200 μm (Table S4). To address this issue, Choi et al. implemented an additional sieving step, which effectively eliminated larger particles.^10^ A reduction in overall particle sizes was either achieved by milling in wet media,^34^ liquid nitrogen cooling,^8,33^ or extensive milling times.^34^ In some instances, researchers also attempted to influence the final MP characteristics through the precursor material chosen such as broken-down single-use plastic,^9,10^ or microbeads.^34^ In comparison, using MFs as a precursor, the particle size, size distribution, and milling time could be drastically reduced without the need for liquid nitrogen cooling. Moreover, the production technique allows for the generation of irregular fragments and fibrous particles by using the same precursor material. In addition, this approach enables the direct embedding of optical markers, such as fluorophores and UCNPs, into the MP, eliminating the need for an additional production step.^8–10^

### 3.3 Application of True-To-Life Microplastic in an *Ex Vivo* Kidney Model

To demonstrate the applicability and detectability of the labeled MPs produced from optimized ball milling, PS-DPA MP was infused into a murine kidney using the MIPK model. This model was chosen since it enables the introduction of the material of interest directly into an intact organ without being dependent on intestinal absorption. Therefore, the MIPK allows one to administer a defined dose to a functional kidney. The fluorescence immunohistochemical (IHC) staining of the PS-DPA MP-perfused kidney included a Phalloidin (label: 488 nm) staining for f-actin, a component of the brush border of proximal tubules, smooth muscle in renal vessels, and glomeruli.^82^ Moreover, the tissue sections were stained for F4/80 (label: 555 nm) as a marker for murine macrophages^83^ and CD31 (label: 647 nm) as an endothelial cell marker.^84^

During microscopic analysis of the MIPK tissue sections, the PS-DPA MPs were successfully detected using the 405 nm laser setup with low laser power (2.88 mW, equals 2.0% max. power). The particles were found in glomeruli and in renal blood vessels with good demarcation from the surrounding tissue (Figures 6 and S15). Most importantly, no overlap was seen between any of the fluorescent antibody signals excited with different lasers, and the emission signal of the PS-DPA MP, confirming the success of the initial labeled MP design criteria. This is relevant as the parallel usage of several antibody stainings facilitates colocalization studies. Furthermore, the low laser power needed for exciting the PS-DPA MP reduces the risk of photobleaching and thermal tissue damage, even after extended exposure times.

**Figure 6:**
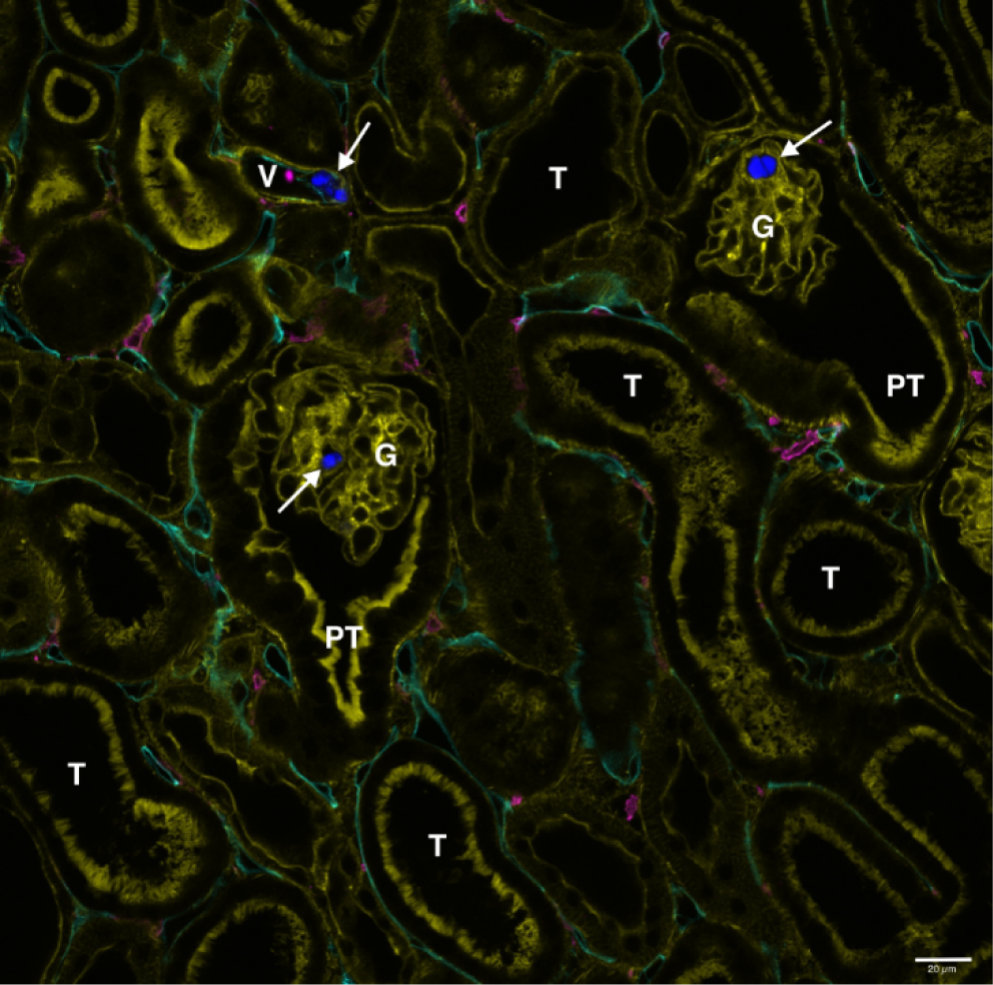
Mouse kidney tissue section (150 μm) of a MIPK, perfused with 0.5 mg of PS-DPA MP (arrows; blue, 405 nm) after fluorescence immunohistochemistry staining for CD31 (endothelial cells; cyan, 647 nm), F4/80 (murine macrophages; magenta, 555 nm), and Phalloidin (f-actin; yellow, 488 nm). Scale bar = 20 μm. G = glomerulus, PT = proximal tubule, T = tubule, V = blood vessel.

Albeit not the focus of this study already, it is noteworthy that the irregularly shaped MPs were found to be stuck in smaller renal vessels and in the glomerular capillaries. Consequently, MP fragments in the vasculature may impair renal blood flow and compromise glomerular filtration, confirming the hypothesis that their study is of high relevance in the understanding of the biotoxicity of environmental MPs.

The goal of this study was to produce optically labeled MPs that more closely resemble the shape of MPs found in the environment. Here, we demonstrated that microfibrous precursor material can easily be used to generate irregularly shaped MPs with a two-step milling approach. As shown in this study, MFs can easily be doped with desirable probes, such as organic and inorganic luminophores. The chosen labels, DPA and (Yb,Tm)-doped UCNPs, do not interfere with necessary staining in cell and histological studies, and MPs from environmental sources might be clearly distinguishable when compared to our produced MPs. As this study demonstrated the simplicity of the stable embedding of molecules and NPs, this method could be further extended to other labels, such as carbon or quantum dots or differently doped rare earth (RE) NPs. The latter can be, *e.g.*, doped with Gd ions, offering the application in magnetic resonance (MR) imaging.^85^ With the low abundance of RE in the environment,^86^ they may also be suitable dopants to enable very sensitive quantitative studies by using elemental detection methods such as ICP-OES or ICP-MS. This would allow for the detection of below 1 μg of plastic (calculated for the doping amount in this study).^57^ Furthermore, simultaneous incorporation of multiple labels might be possible, enabling the multimodal characterization of MPs, combining qualitative and quantitative methods.

In this study, pristine PS was used as a model polymer due to its ubiquity in MP research. However, MFs can be produced from a variety of polymers. Subsequent research could, therefore, adapt the protocol for different types of MPs and further optimize the production of fibrous MPs. Also, MPs found in the environment undergo several different degradation processes, such as UV exposure or biofilm formation.^87,88^ This should be simulated in future research, where our labeled MPs could enable tailored investigations of aging processes, *e.g.*, by using UCNPs as labels for UV weathering.

Based on this work, future studies will focus on biological interactions such as uptake and distribution, simulation of environmental aging, and expanding the method to other polymers. Our aim is to support the development of standardized model particles for MP research, serving as reliable tracer materials closer to environmental MP.

## Supplementary Material



## Notes

Detailed information about the fluorophores used, laser-cutting of the knife-coated polymer foils, spectra of the resulting polymer sheets doped with the fluorophores, the appearance of the polymer foils under a fluorescent microscope, a concentration dependency study of the fluorophores, synthesis and characterization of the UCNPs including TEM, DLS and luminescence characterization, SEM images of UCNP-doped MFs and MP particles, further information on the microscopic evaluation of the UCNP-doped samples, SEM images of PS MFs doped and nondoped, of the MP produced with the different methods discussed in the paper, and FT-IR spectra of PS during the MP production steps (PDF)
