# Supplementary material for: Production of Irregularly Shaped True-To-Life Microplastics with Embedded Optical Labels and Exemplary Application in an Ex Vivo Model
Source: Environ Sci Technol. 2025 Aug 28;59(35):18525–37. doi: 10.1021/acs.est.5c08586 (PMC12424183; doi:10.1021/acs.est.5c08586)
Supplement: Supplementary file 1 [file es5c08586_si_001.pdf]

SUPPORTING INFORMATION

Production of Irregularly Shaped True-to-life Microplastics with Embedded Optical Labels and Exemplary Application in an *ex vivo* Model

*Alissa J. Wieberneit[a]‡, Sophia J. Baumann[a]‡, Hannah Triebel[b]‡, Sarah Dietrich[a], Nongnoot Wongkaew[a], Hayo Castrop[b], Antje J. Baeumner[a]**

[a]Institute of Analytical Chemistry, Chemo- and Biosensors, University of Regensburg, Universitaetsstrasse 31, 93053 Regensburg, Germany

[b]Institute of Physiology, University of Regensburg, Universitaetsstrasse 31, 93053 Regensburg, Germany

‡ A.J.W., S.J.B., H.T. contributed equally to this work

* Corresponding author: antje.baeumner@ur.de

Summary of Supporting Information:

Number of pages	22
Number of tables	4
Number of figures	15

1 MATERIALS AND METHODS

1.1 LIST OF CHEMICALS

If not stated in the text, chemicals and suppliers are listed in the following:

Agarose (low melting) was purchased from Biozym Scientific GmbH (Hessisch Oldendorf, Germany). Ethanol (EtOH, technical grade) was purchased from CSC Jäklechemie GmbH & Co. KG (Nuremberg, Germany). Dimethylformamide (DMF, ≥ 99.5%), cyclohexane (≥ 99.5%), oleic acid (OA, ≥ 90%), 1-octadecene (ODE, ≥ 90%), chloroform ($CHCl_3$, ≥ 99%), methanol (MeOH, ≥ 99.9%), nitric acid (HNO_3, 65% v/v), sulfuric acid (H_2SO_4, 96% v/v) and tetrahydrofuran (THF, ≥ 99.8%) were purchased from Fisher Chemicals (Schwerte, Germany). Sodium hydroxide (NaOH, p.A.), hydrochloric acid (HCl, 1 M), paraformaldehyde and horse serum were purchased from Merck (Darmstadt, Germany). Thulium(III)-chlorid hexahydrate ($TmCl_3 \times 6\ H_2O$, ≥ 99.99%), sodium oleate (Na-OA, ≥ 82%), ammonium fluoride (NH_4F, ≥ 99.99%), nitrosyl tetrafluoroborate ($NOBF_4$, ≥ 95%), ethanol (EtOH, p.A.), bovine serum albumine (BSA), phosphate buffered saline (PBS), polystyrene (PS, MW ≈ 280,000), perylene, diphenylanthracene (DPA), sodium dodecyl sulfate (SDS, ≥ 98.5%), Trizma base (Tris, ≥ 99.9%), MES hydrate (≥ 99.5%), and soy lecithin were purchased from Sigma Aldrich (St. Louis, USA). Sodium chloride (NaCl, ≥ 99.5%) and ethylenediaminetetraacetic acid (EDTA, ≥ 99%) were purchased from Carl Roth GmbH & Co. KG (Karlsruhe, Germany). Saccharose was purchased from Thermo Fisher (Waltham, USA). Ytterbium(III)-chloride hexahydrate ($YbCl_3 \times 6\ H_2O$, ≥ 99.9%) and yttrium(III)-chloride hexahydrate ($YCl_3 \times 6\ H_2O$, ≥ 99.9%) were purchased from Treibacher Industry AG (Althofen, Austria). ICP multielement standard (Er, Gd, Nd, Tm, Y, Yb, 1000 $mg \cdot mL^{-1}$ in 1.5 M HNO_3) was purchased from Bernd Kraft GmbH (Den Haag, Netherlands).

1.2 APPLICATION-RELATED SELECTION OF OPTICAL LABELS

1.2.1 FLOURESCENT LABELLING WITH DPA

Table S 1: Overview of the measurement parameters of the fluorescence spectra with the slit size.

Ex / nm	Slit size / nm	Em start / nm	Em end / nm
405	10	430	700
488	10	510	700
561	10	590	700
633	10	660	700

Table S 2: Comparison of possible fluorophores:

	DPA[1,2]	Perylene[1,2]
Emission maximum / nm	400 – 500	435; 464
Quantum yield	0.95 – 1.05	0.82 – 0.94
Molar absorption coefficient ε	14 000 in cyclohexane	50 000 in chloroform
Costs according to Sigma Aldrich / € / g	46.60	103.00

Detailed Information about Laser-Cutting of the Knife-coated Polymer Foils

In order to laser-cut the foils, the desired designs were drawn as vector graphics with CorelDraw suite 24.0. To work in vector mode, all lines needed to be defined as hairlines. Engraving was performed with a VLS 2.0 laser engraving systems based on a 10 600 nm infrared laser with a maximum power output of 30 W using 5% power and 60% speed for cutting.

1.2.2 UPCONVERSION NANOPARTICLES

Synthesis of Core UCNPs

The synthesis of (Yb,Tm)-doped β-$NaYF_4$ upconversion nanoparticles was followed by a protocol of Schroter et al. with minor modifications.[3] For a 5 mmol batch size, YCl_3×6 H_2O (3.72 mmol, 78%), $YbCl_3$×6 H_2O (1.25 mmol, 25%), and $TmCl_3$×6 H_2O (0.015 mmol, 0.3%) were dispersed in methanol. The solution was transferred to a three-necked round-bottom flask under nitrogen atmosphere. Then, oleic acid (30 mL) and 1-octadecene (50 mL) were slowly added. The mixture was heated to 110 °C, and vacuum was applied for one hour. Afterwards, the reaction solution was cooled to room temperature, and NH_4F (20 mmol), Na-oleate (15.65 mmol), and 1-octadecene (25 mL) were added. Vacuum was applied for 50 min, followed by degassing of the solution three times. The solution was then heated to 315 °C with a heating rate of 16 °C per minute and kept under reflux for 20 min, followed by rapid cooling to room temperature. For purification, the reaction mixture was first collected in centrifuge tubes by precipitation with an excess of ethanol and centrifuged at 3850 *g* (10 min) with a Hettich Universal 320R centrifuge. The resulting pellets were redispersed in a small amount of cyclohexane, precipitated again with excess of ethanol, and collected by centrifugation (3850 *g*, 10 min). This process was repeated twice. Finally, the particles were redispersed in cyclohexane (30 mL) and aggregates were removed by centrifugation (1200 *g*, 5 min). The final particle dispersion was stored at 8 °C until further use.[4]

Synthesis of Shell Precursors[3]

In a 10 mmol batch size (regarding the lanthanide content) for cubic α-$NaYF_4$ particles as shell precursors, YCl_3×6 H_2O was dissolved methanol and added to a three-necked round-bottom flask under nitrogen atmosphere. Oleic acid (80 mL) and 1-octadecene (150 mL) were slowly added. The mixture was heated to 160 °C, and vacuum was applied for 30 min. After cooling to room temperature, NH_4F (40 mmol) and NaOH (25 mmol, dissolved in MeOH) were added. The solution was heated to 120 °C and kept at this temperature for further 30 min. Afterwards, the reaction mixture was heated to 240 °C (16 $°C \cdot min^{-1}$) under reflux for 30 min. The solution was rapidly cooled to room temperature and purification was performed, as described for the core particles.

Core-shell Synthesis[3]

For a shell thickness of approximately 3 nm, core particles dispersed in cyclohexane (1 mmol regarding the total lanthanide content), oleic acid (5 mL), and 1-octadecene (5 mL) were added to a three-necked round bottom flask and heated to 100 °C under constant nitrogen flow. In a second flask, the α-$NaYF_4$ particles (1.7 mmol, regarding the total lanthanide content) dispersed in cyclohexane, oleic acid (8.55 mL), and 1-octadecene (8.55 mL) were also heated to 100 °C. After applying vacuum to both flasks for one hour, the flask containing the core particles was heated to 315 °C (16 °C·min^{-1}) under nitrogen atmosphere. Purification of the particles was performed as described above.

Surface Modification of UCNPs for Dispersion in DMF:THF

The protocol for the surface modification of UCNPs for the dispersion in a DMF:THF mixture was adapted from Himmelstoß et al.[4] For a 100 mg batch size (mass UCNP), the particles dispersed in cyclohexane were added to a round-bottom flask, and DMF (6 mL) was slowly added under constant stirring. The mixture was heated to 30 °C for 10 min. Then, $NOBF_4$ (150 mg) was added, and the temperature was increased to 60 °C. The stirring speed was increased to 1200 rpm and the reaction was held at elevated temperatures for 30 min. Afterwards, the solutions was ultrasonicated for a few minutes. For purification the particles were precipitated from the cyclohexane-DMF mixture by adding an excess of chloroform, followed by centrifugation for 15 min at 2000 *g*. The resulting pellet was redispersed in a 1:1 DMF:THF mixture.

Microscopic Evaluation for UCNP-doped Materials

Despite the benefit of an additional antibody staining wavelength due to the change of the excitation wavelength to 980 nm, the visualization of UCNP-doped MPs has some disadvantages. Standard fluorescent microscopes are often not equipped with a NIR or 2-photon laser. Apart from that, common microscopy setups might struggle with the long luminescence lifetime of the UCNPs since some detectors collect light from the entire sample and assign it to the excitation point which

could result in a blurred or streaky image.[5] To address these problems, a very slow scanning time was used.

1.3 CHARACTERIZATION

SEM and TEM micrographs analysis was conducted using ImageJ (Fiji, v. 1.5.4f) and Origin (Version 2022b). For SEM analysis, the following steps were conducted: The micrographs were calibrated using the image pixel size given in each image. Only images of the same magnification were used for the evaluation. For fibers, only sharply focused fibers in the focal plane were used for evaluation. Here, the shortest diameter between the edges was measured with the straight-line tool in ImageJ at multiple positions along each fiber to capture thickness fluctuations. To determine the diameter of milled MP samples, again the straight-line tool was used to measure the diameter of each particle. For fibrous particles, the length of the particle was evaluated with the segmented-line tool in ImageJ as the diameter of the particles is defined by the thickness of the MF.

For TEM micrograph analysis, the following steps were conducted: The scale is stored in the image format, so no calibration was necessary. The particle diameter was determined using the plugin “ParticleSizer” by Thorsten Wagner. Additionally, for ParticleSizer to function properly, the plugins “BioVoxxel” and “Biomedgroup” were loaded. In the settings menu of the ParticleSizer plugin, the following settings were activated: “Use watershed for irregular structures”, with the convexity threshold varied between 0.9 and 1, depending on the amount of particle overlay, “Use inverted images”, “Ask me to select a region”, “Do not plot size distribution”, and “Record process” (under misc). All other settings were used as default. Then, the plugin is applied for several micrographs, were the region of interest (whole micrograph) is selected.

For both, TEM and SEM analysis, the measured data was imported to Origin. Here, the Feret diameter is plotted as histogram with corresponding size distribution. The mean diameter, the standard deviation and the number of particles/fibers measured were determined using the “Statistics on Column” feature of Origin, in default settings.

2 SUPPLEMENTARY DATA AND FIGURES

2.1 APPLICATION-RELATED SELECTION OF OPTICAL LABELS

2.1.1 FLOURESCENT LABELLING WITH DPA

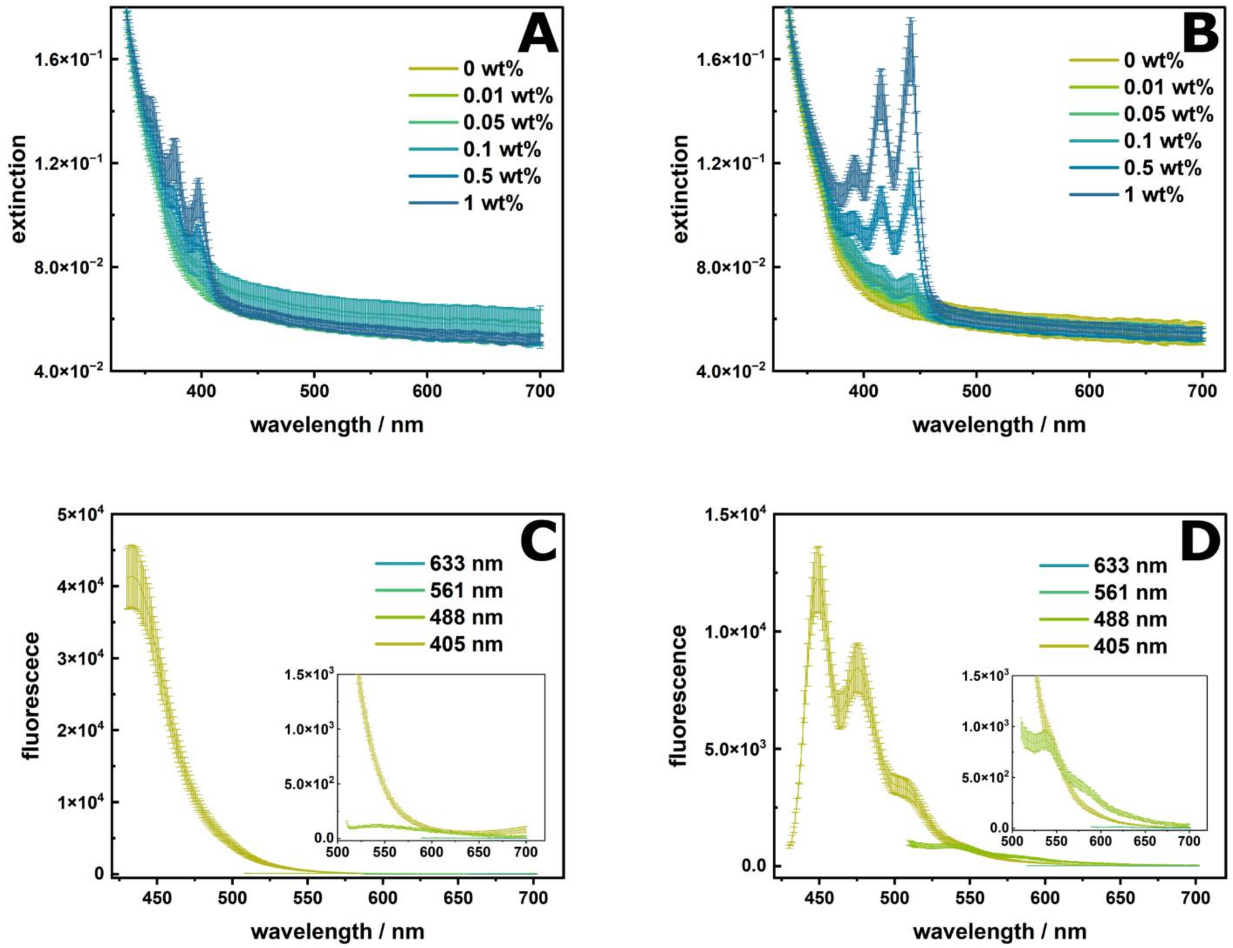

Figure S 1: Extinction and fluorescence spectra of polymer foils from Figure S2 (A,B) Extinction spectra in dependency of doping concentration of DPA (A) and perylene (B). (C,D) Fluorescence spectra of 1 wt% doping of DPA (C) and perylene (D) excited with different wavelengths.

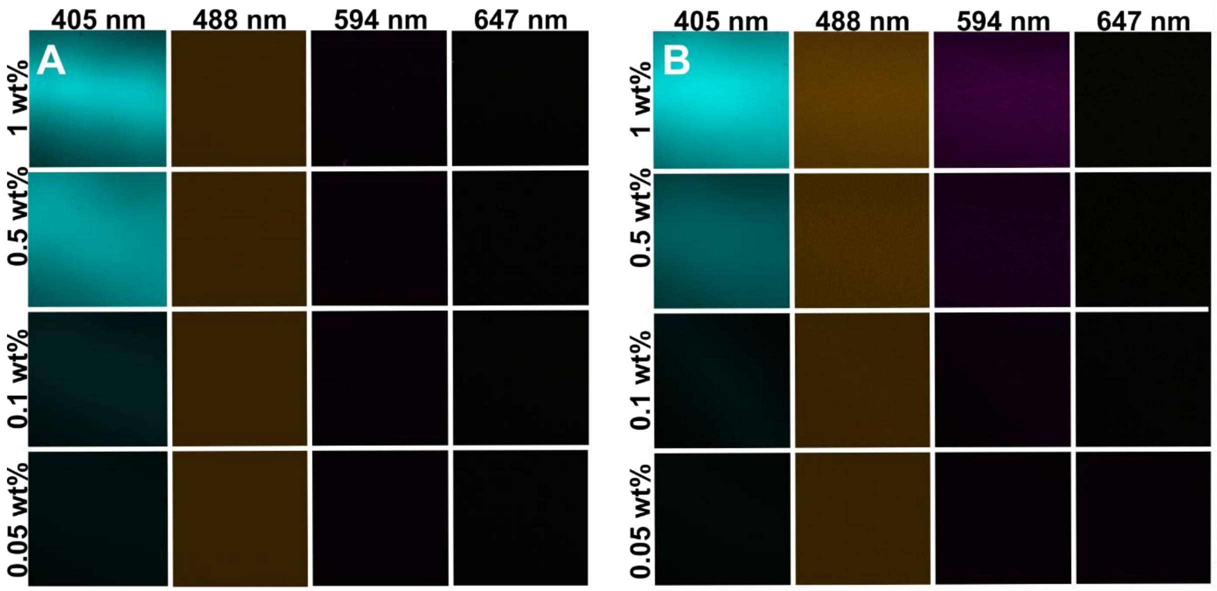

Figure S 2: Appearance of knife coated polymer foils with various doping concentration under the fluorescent microscope under all wavelengths used for histological analysis. (A) DPA and (B) perylene. Gain was optimized to highest concentration for each fluorophore individually.

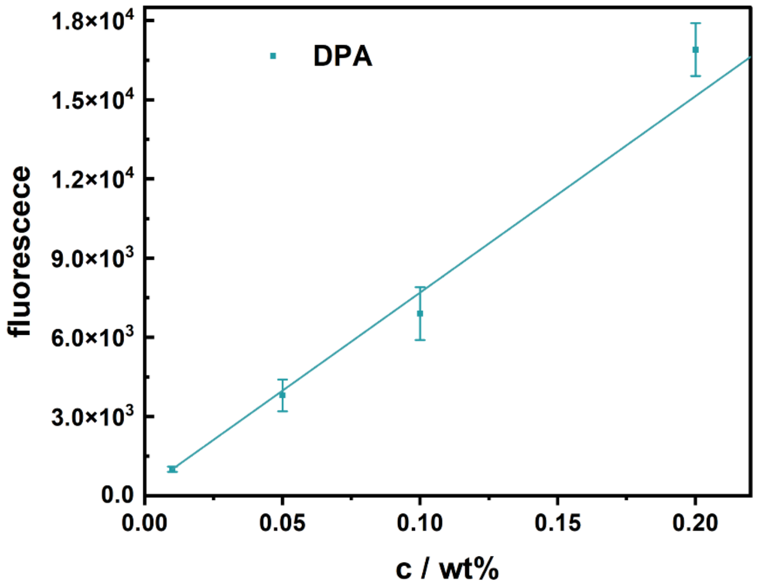

Figure S 3: Zoom-in to Figure 1C. Dependency of fluorescence signal to the doping concentration of the fluorophore. The maximum fluorescence signal was plotted against the doping ratio, with a linear correlation up to 0.6 wt% (R^2 DPA: 0.993), λ_{ex}: 405 nm. Measurements done with knife coated polymer sheets (n ≥ 20).

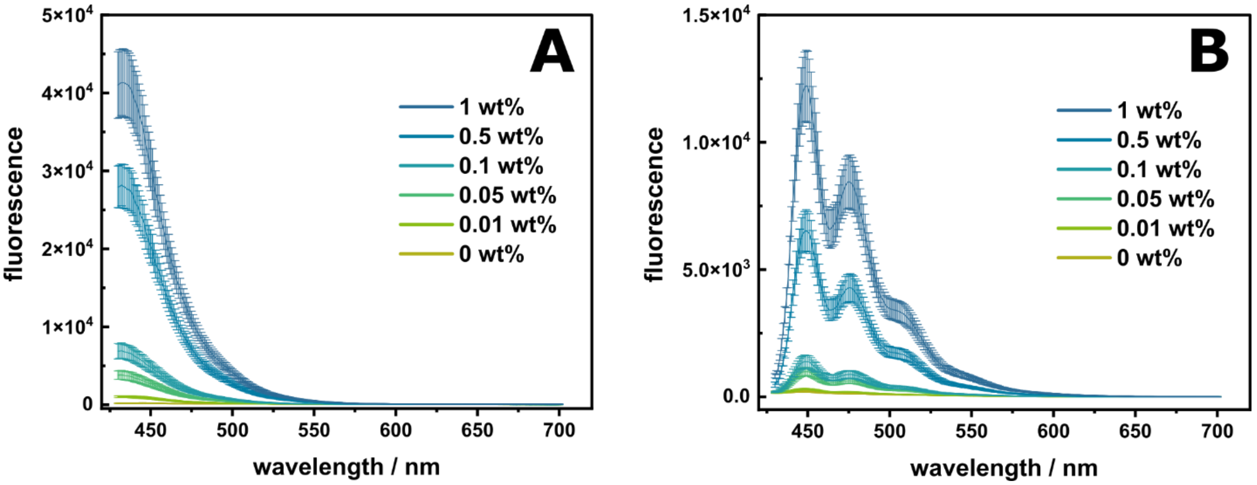

Figure S 4: Concentration dependency of the doping concentration on the fluorescent spectra of knife coated PS-sheets (λ_{ex}: 405 nm). (A) DPA, (B) perylene.

2.1.2 UPCONVERSION NANOPARTICLES

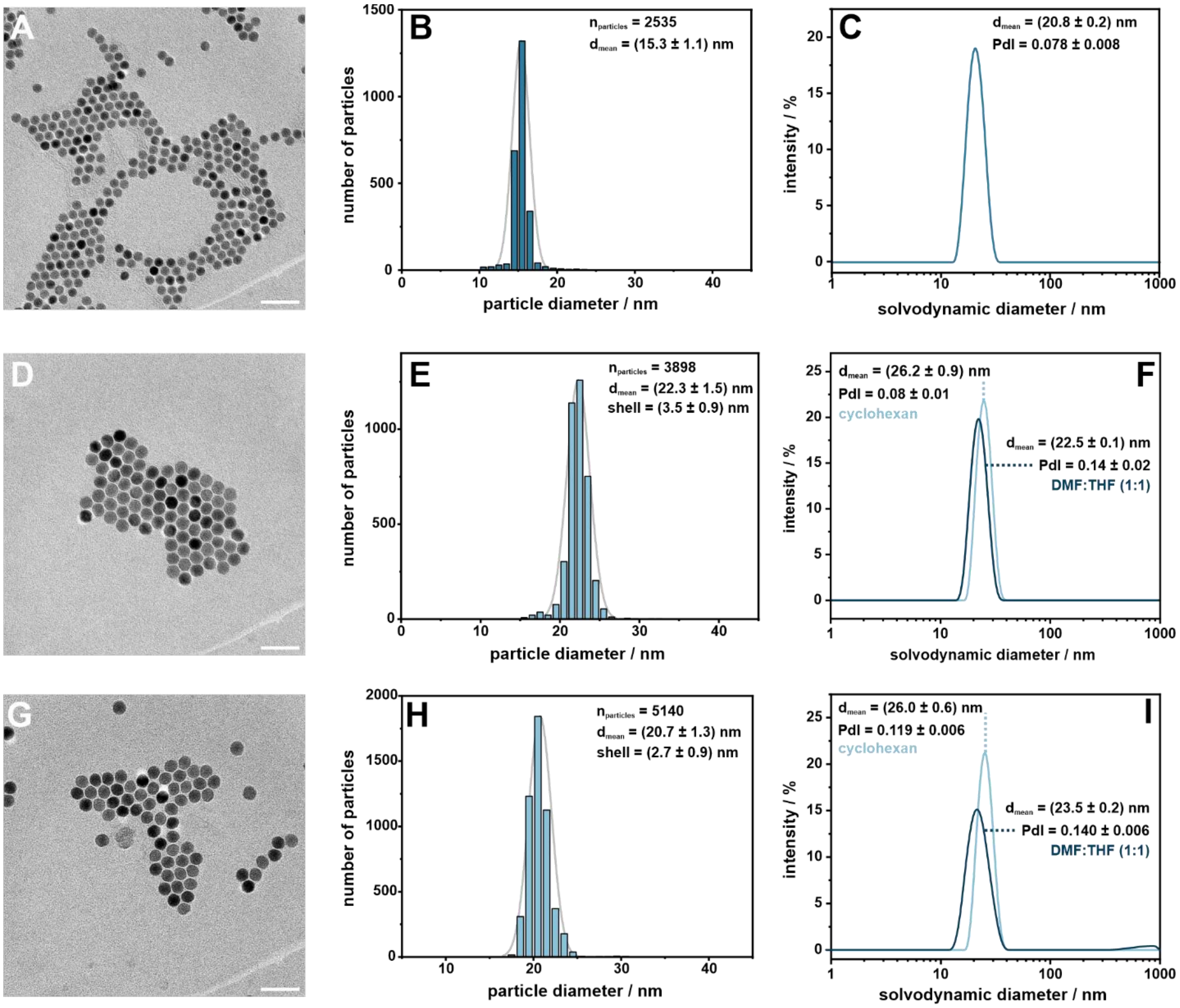

Figure S 5: Characterization of UCNPs used as dopant in PS MFs. (A,B,C) TEM micrograph with corresponding size distribution histogram and DLS in cyclohexane of $NaYF_4$:Yb,Tm doped core particles. TEM analysis results in a diameter of (15.3 ± 1.1) nm. The solvodynamic diameter is determined to (20.8 ± 0.2) nm with a PdI of 0.078 ± 0.008 (n = 3). (D,E,F) TEM micrograph with corresponding size distribution histogram and DLS in cyclohexane (light blue) and in DMF:THF (dark blue) of $NaYF_4$:Yb,Tm@$NaYF_4$ doped core-shell particles (first batch). TEM analysis results in a diameter of (22.3 ± 1.5) nm, corresponding to a shell thickness of (3.5 ± 0.9) nm. The solvodynamic diameter is determined to (26.2 ± 0.9) nm with a PdI of 0.08 ± 0.01 (n=3). For the particles in DMF:THF the solvodynamic diameter is determined to (22.5 ± 0.1) nm with a PdI of 0.14 ± 0.02 (n = 3). (G,H,I) TEM micrograph with corresponding size distribution histogram and DLS in cyclohexane (light blue) and in DMF:THF (dark blue) of $NaYF_4$:Yb,Tm@$NaYF_4$ doped

core-shell particles (second batch). TEM analysis results in a diameter of (20.7 ± 1.3) nm, corresponding to a shell thickness of (2.7 ± 0.9) nm. The solvodynamic diameter in cyclohexane is determined to (26.0 ± 0.6) nm with a PdI of 0.119 ± 0.006 (n = 3). For the particles in DMF:THF the solvodynamic diameter is determined to (23.5 ± 0.2) nm with a PdI of 0.140 ± 0.006 (n = 3). The scale bar in the TEM micrographs corresponds to 100 nm.

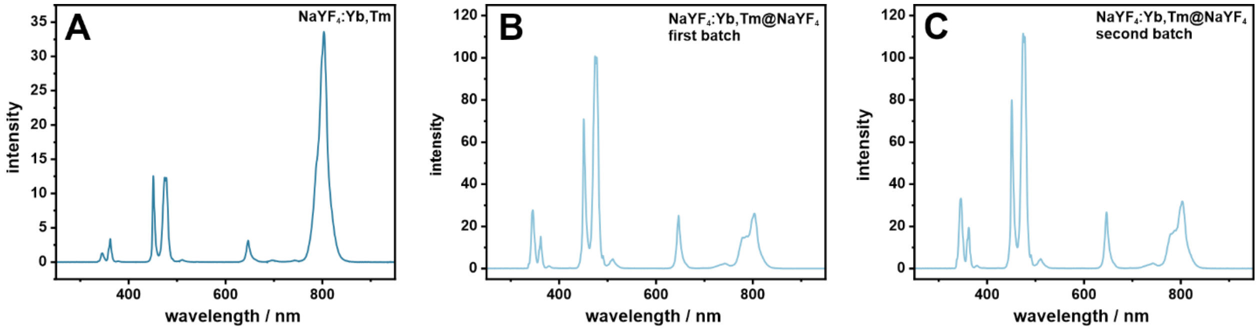

Figure S 6: Luminescence characterization of UCNPs used as dopant in PS MFs. (A) Luminescence spectra of $NaYF_4$:Yb,Tm doped core particles in cyclohexane. (B) Luminescence spectra of the first batch of $NaYF_4$:Yb,Tm@$NaYF_4$ doped core-shell particles in cyclohexane. (C) Luminescence spectra of the second batch of $NaYF_4$:Yb,Tm@$NaYF_4$ doped core-shell particles in cyclohexane. Spectra are normalized to the respective Yb^{3+}-concentration. Excitation with λ_{ex} = 980 nm, 150 W·cm^{-2}.

Characterization of UCNPs

TEM measurements show uniform spherical particles with a narrow size distribution for the core and both core-shell particles batches (Figure S5). The core-shell particles have a shell thickness of about 3 nm and are comparable within the range of the standard deviation (first batch: (3.5 ± 0.9) nm, second batch: (2.7 ± 0.9) nm). DLS measurements (Figure S5C, F, I) support the results from TEM measurement, showing a small polydispersity index around 0.1, which indicates high uniformity and colloidal stability of the particles.

For embedding into PS spinning solutions, the particles were transferred into DMF by a ligand removal reaction.[4,6] After addition of THF, the particles were still colloidally stable without any

formation of agglomerates or a sign of sedimentation as indicated by the small PdI, which is important for a homogeneous distribution of the particles in the MFs (Figure S5F, I).

Luminescence spectra have been recorded to characterize the luminescence properties of the UCNPs. Tm-doped nanoparticles show emissions in the UV, blue, red, and NIR region. By equipping the core particles (Figure S6A), with an optically silent $NaYF_4$ shell, the overall luminescence intensity can be drastically increase (Figure S6B, C), and the ratio of the emissions is shifted towards the blue emission (≈ 450 – 490 nm).

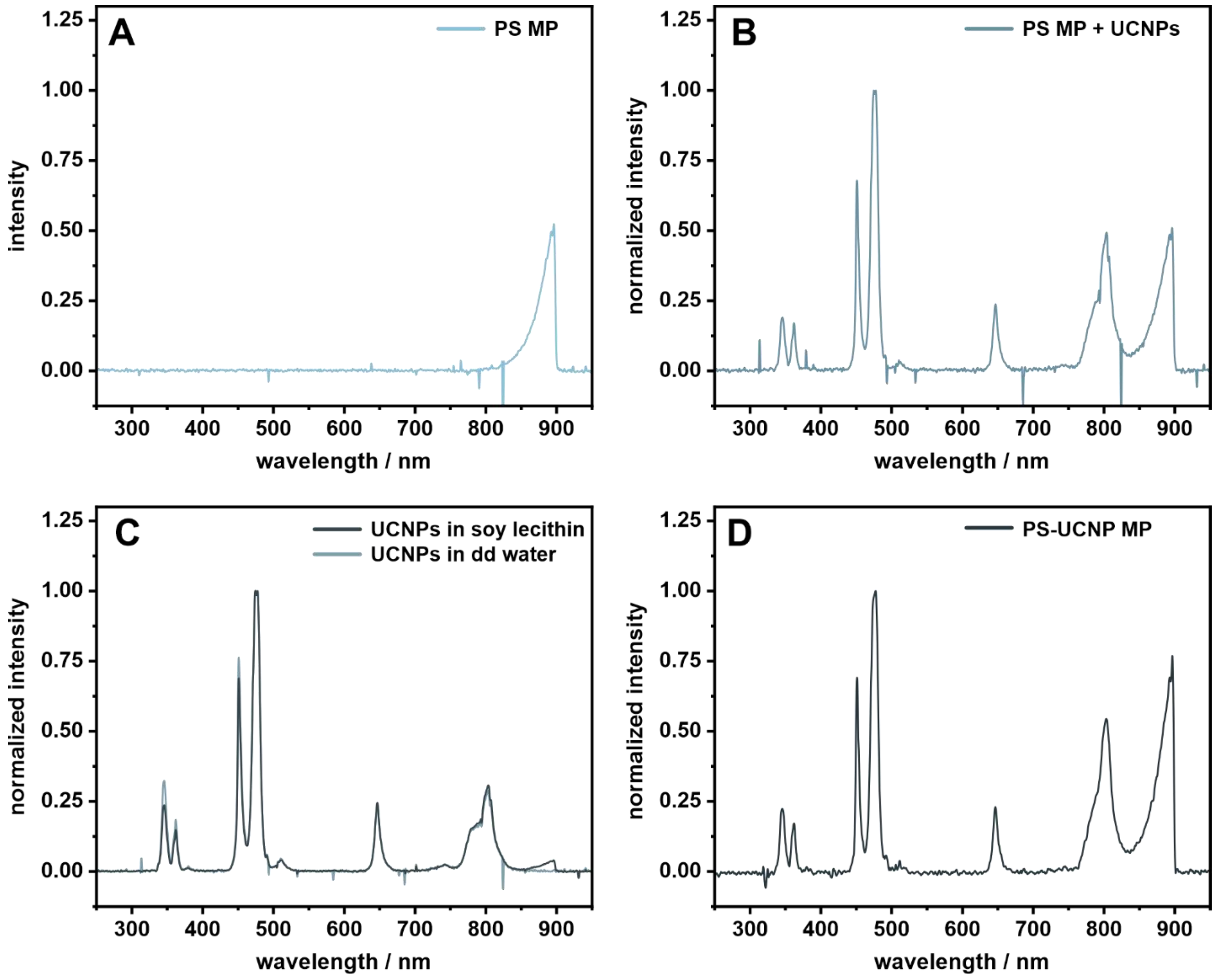

Figure S 7: Control experiments for the luminescence characterization of UCNPs in PS MP. (A) PS MP without UCNPs. (B) PS MP with the addition of UCNPs. (C) UCNPs in soy lecithin (1 wt%, 1:10 dilution) and double-distilled (dd) water. (D) PS-UCNP MP. If not stated otherwise, all measurements have been performed in a 1 wt% soy lecithin solution (1:10 dilution,

$\beta_{initial}$(MP) = 4 mg·mL^{-1}). All spectra containing UCNPs were normalized to the 477 nm emission (λ_{ex} = 980 nm, 150 W·cm^{-2}, cw).

2.2 MICROFIBERS AS PRECURSOR FOR THE PRODUCTION OF TRUE-TO-LIFE MICROPLASTIC

2.2.1 STABLE EMBEDDING OF OPTICAL LABELS IN POLYMER MATRIX

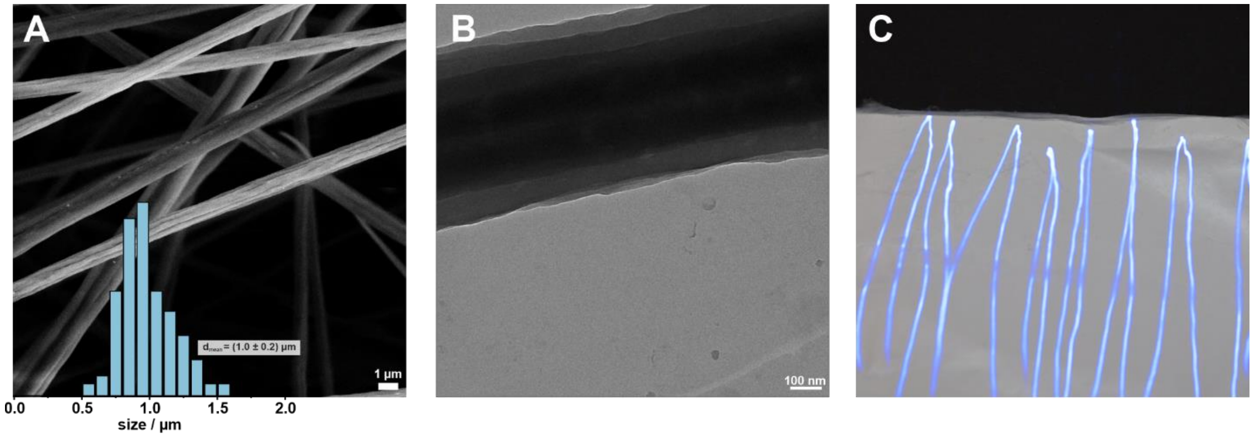

Figure S 8: Characterization of PS MFs doped with UCNPs. (A) SEM micrographs showing an overview of the PS-UCNP MFs. SEM analysis results in a diameter of (1.0 ± 0.2) µm, the scale bar corresponds to 1 µm. (B) TEM micrograph of one single fiber. Scale bar corresponds to 100 nm. (C) Merged photograph of MF mat doped with UCNPs, excited with a handheld laser module (λ_{ex} = 980 nm, 350 mW, cw).

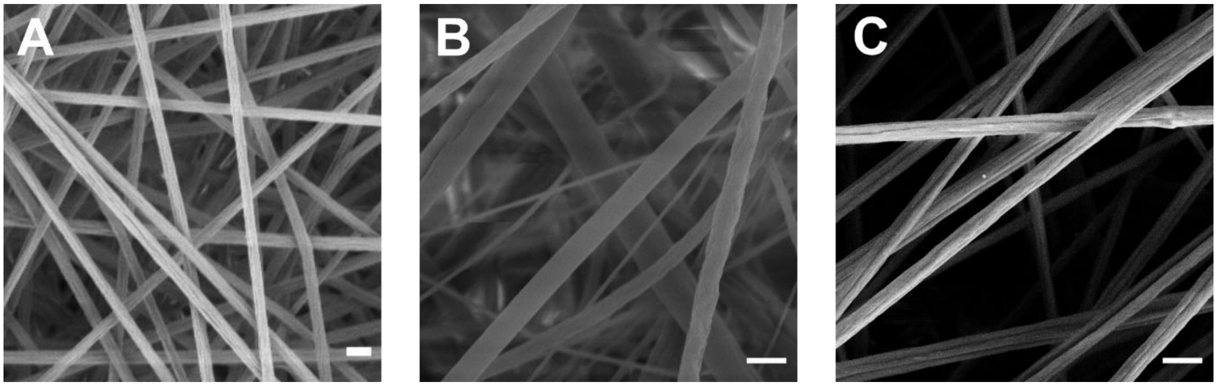

Figure S 9: SEM images of the PS MFs with different doping, scale bar 2 µm. (A) PS MFs, (B) PS-DPA MFs, (C) PS-UCNP MFs.

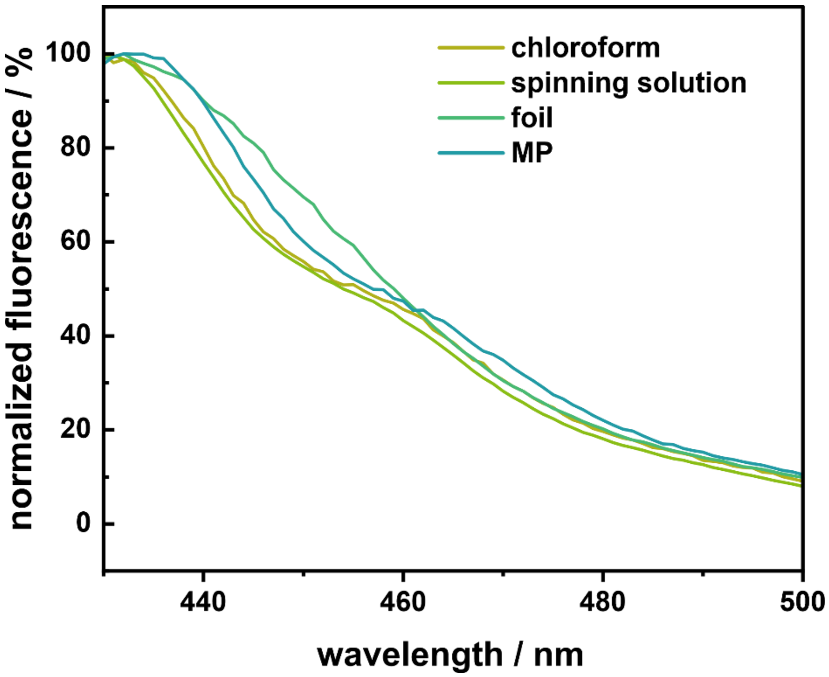

Figure S 10: Fluorescence spectra of DPA in chloroform, in the spinning solution, in knife-coated polymer foils, and in MP stabilized in a 1 wt% soy lecithin solution. λ_{ex} = 405 nm, step size 1 nm. Fluorescence signal normalized to the maximum intensity.

Calculation of Molar Concentration of Fluoride Released from the PS-UCNP MFs

$$n(UCNP) = \frac{m(UCNP)}{M(UCNP)} \tag{1}$$

$$NaYF_4 \rightleftharpoons Na^+ + Y^{3+} + 4F^- \tag{2}$$

$$n(F^-) = 4 \cdot n(UCNP) \tag{3}$$

$$c(F^-) = n(F^-) \cdot V(total) \tag{4}$$

With *n(UCNP)* being the molar amount of substance, *m(UCNP)* the mass of UCNPs leached from the MFs derived from ICP-OES measurements, *M(UCNP)* the theoretical molar mass of the UCNPs, *n(F^-)* the molar amount of substance of fluoride ions, *c(F^-)* the molar concentration of fluoride ions, and *V(total)* the total volume used in the leaching study (560 mL). Equation (2) shows the law of mass action for $NaYF_4$.

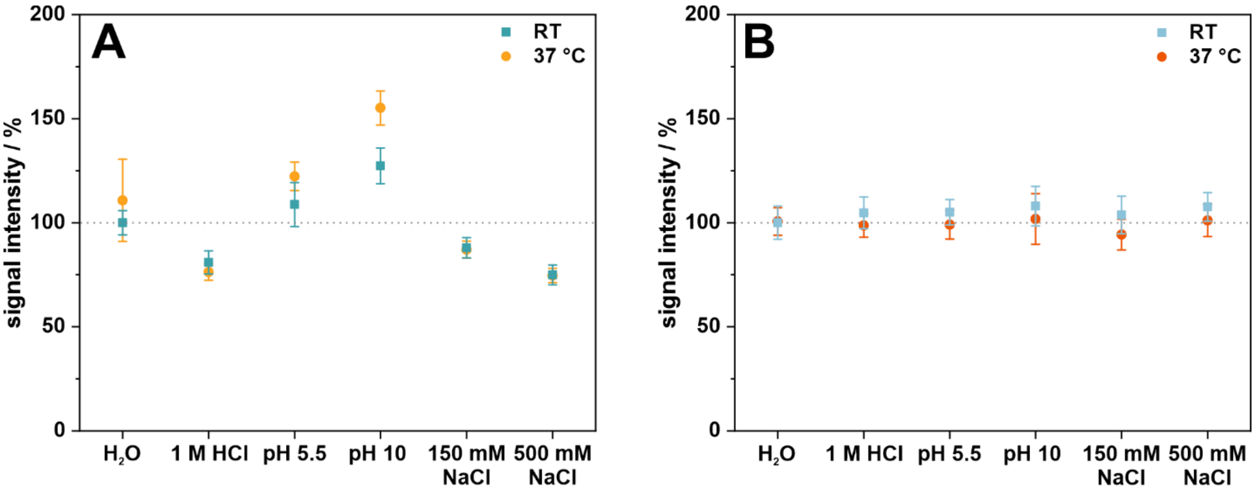

Figure S 11: Luminescence measurements under various environmental conditions (water, 1 M HCl, pH 5.5, pH 10, 150 mM NaCl, 500 mM NaCl; $\beta_{initial}(MP) = 4\ mg \cdot mL^{-1}$, 1:10 dilution, $n \geq 3$) of PS-DPA MP (A) and PS-UCNP MP (B). For PS-DPA MP, the fluorescence emission was recorded at $\lambda_{ex} = 405$ nm, $\lambda_{em} = 434$ nm. PS-UCNP MP spectra were normalized to the 477 nm emission ($\lambda_{ex} = 980$ nm, integration area 308 – 837 nm, 150 $W \cdot cm^{-2}$, cw). The luminescence of both was normalized to the signal determined in water at RT. The grey dotted line at 100% serves as a visual reference.

2.2.2 PRODUCTION OF MICROPLASTIC WITH CONTROL OF PARTICLE SHAPE

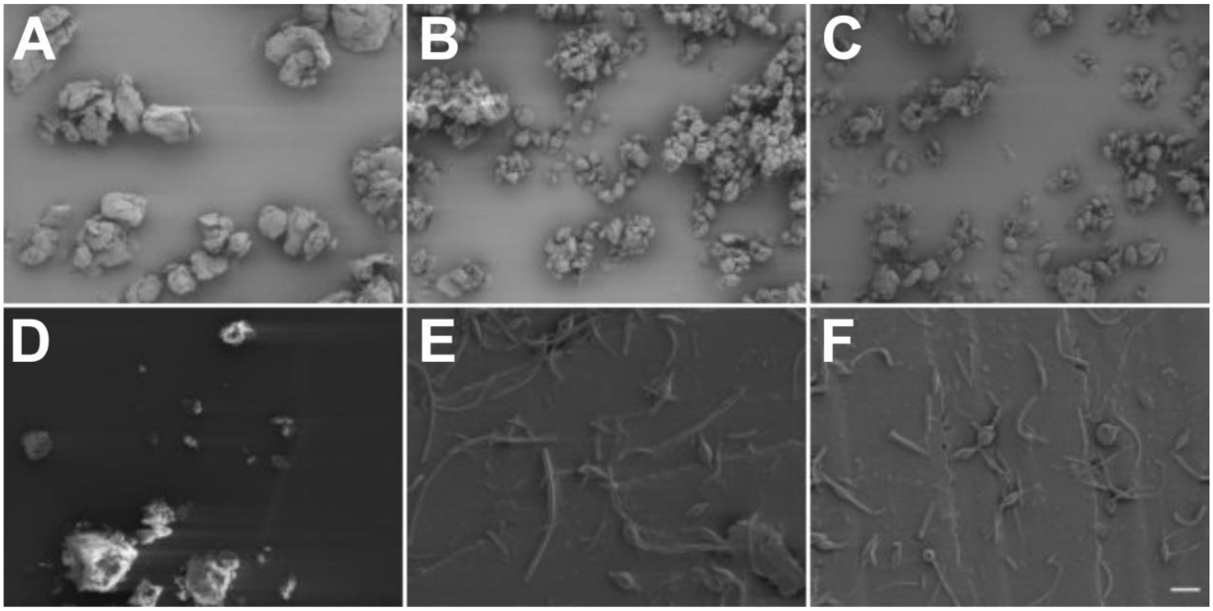

Figure S 12: SEM images with a magnification of 1000 of the microplastic produced by different methods, scale bar 20 µm. (A) Microplastic generated by a BM (50 mL grinding jar; 7× 10 mm grinding balls) after 9 milling steps of 2 min. (B) Microplastic generated by a BM (50 mL grinding jar, about 3000× 2 mm grinding balls) after 9 milling steps of 2 min. (C) Particles from (A) after a second milling step with 9 cycles of each 2 min with smaller balls (50 mL grinding jar, about 3000× 2 mm grinding balls). (D) Particles obtained from a cryo mill under liquid nitrogen cooling (50 mL grinding jar, 8× 12 mm grinding balls) after 9 milling cycles of 2 min with 30 s cooling steps. (E) Particles produced by an ultraturrax in 1 wt% soy lecithin solution before and (F) after filtration through a 20 µm metal sieve.

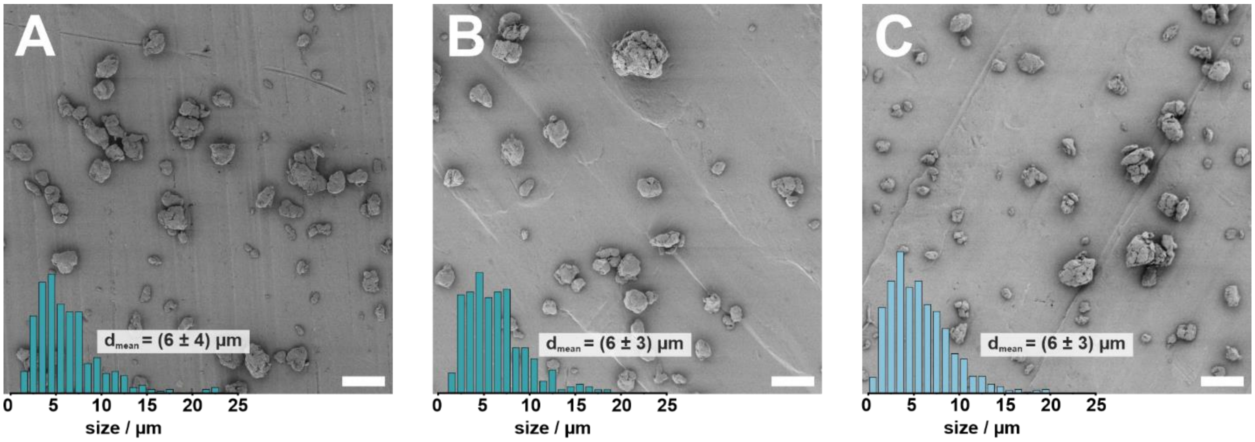

Figure S 13: SEM images with corresponding size distribution analysis of differently doped PS MP produced with the optimized ball milling protocol. (A) undoped PS MP with a diameter of (6 ± 4) µm. (B) second batch of PS-DPA MP with a diameter of (6 ± 3) µm (C) PS-UCNP MP with a diameter of (6 ± 3) µm. The scale bar corresponds to 20 µm; n > 250.

Table S3: Zeta potential measurements of PS MP, PS-DPA MP and PS-UCNP MP in 10 mM KNO_3 (β_{MP} = 0.5 mg·mL^{-1}).

	PS MP	PS-DPA MP	PS-UCNP MP
Zeta potential / mV	-33.2 ± 1.0	-34 ± 2	-27.8 ± 0.9

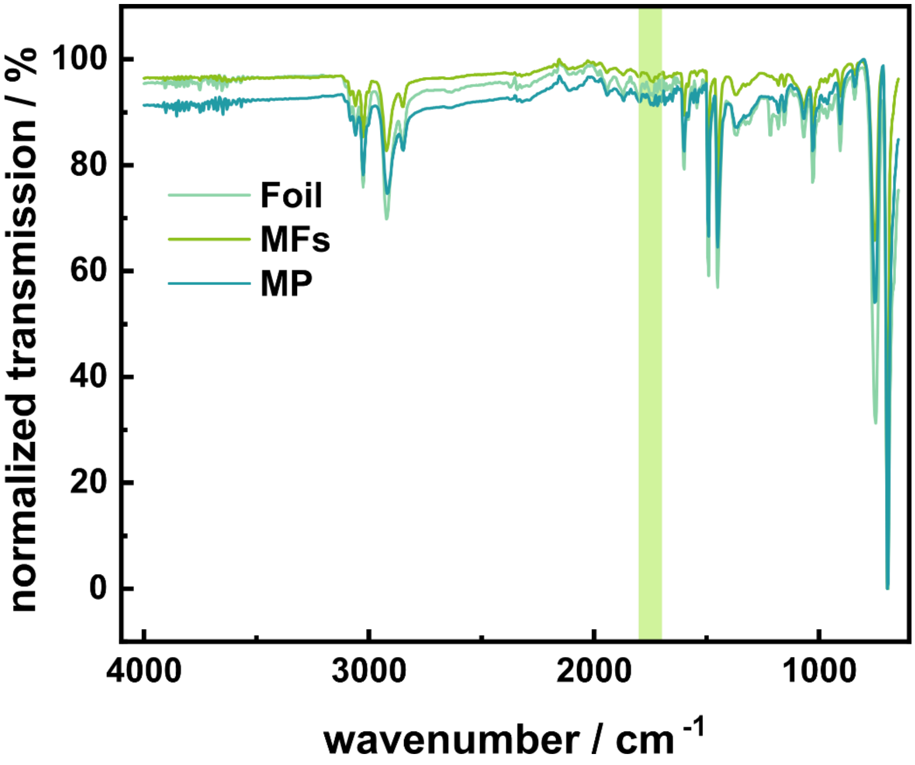

Figure S 14: FT-IR spectra of PS during different steps of the MP production: knife-coated polymer foils, electrospun MFs and MP produced by ball milling. The green bar indicates wavelengths for potential oxidation peaks.[7] The spectra were recorded using the Cary 630 FTIR spectrometer (Agilent) and analyzed with the software MicroLab (Agilent).

Table S 4: Comparison of artificial PS MP by top-down approaches.

Material	Final Size / µm	Production method	Medium	T / °C	Production time / h	Shape	Label	Ref
**Shredded single use products**	1 – 3	Lab-based weathering	Air	ambient	16	Rough surface, heterogenous fragments	none	[8]
**Pellets**	1 – 200	Cryo Milling	Air	Liquid nitrogen	3	Rough surface, heterogenous fragments	none	[9]
**Polymer Beads**	40 – 500	Cryo Milling	Air	Liquid nitrogen	Not given	Rough surface, heterogenous fragments	none	[10]
**250 – 500 µm**	> 5	Stirred Wet Milling	Denatured ethanol, n-hexane	ambient	22	Not shown	none	[11]
**squares (1 cm^2)**	0.1 – 1000	Sonication	0.25 M KOH	ambient	15	Irregular fragments and films with rough surface	none	[12]
**Smashed pellets**	5 – 25	Ball Milling and sieving	Air	cooled	2	Rough surface, heterogenous fragments	none	[13]
**MFs 950 nm**	20 ± 20	Ultraturrax	Soy lecithin	0 °C	1	fibrous	Fluorophores UCNPs	This work
	4 ± 3	Ball Milling	Air	ambient	1.5	Rough surface, heterogenous fragments		

2.3 APPLICATION OF TRUE-TO-LIFE MICROPLASTICS IN AN *EX VIVO* KIDNEY MODEL

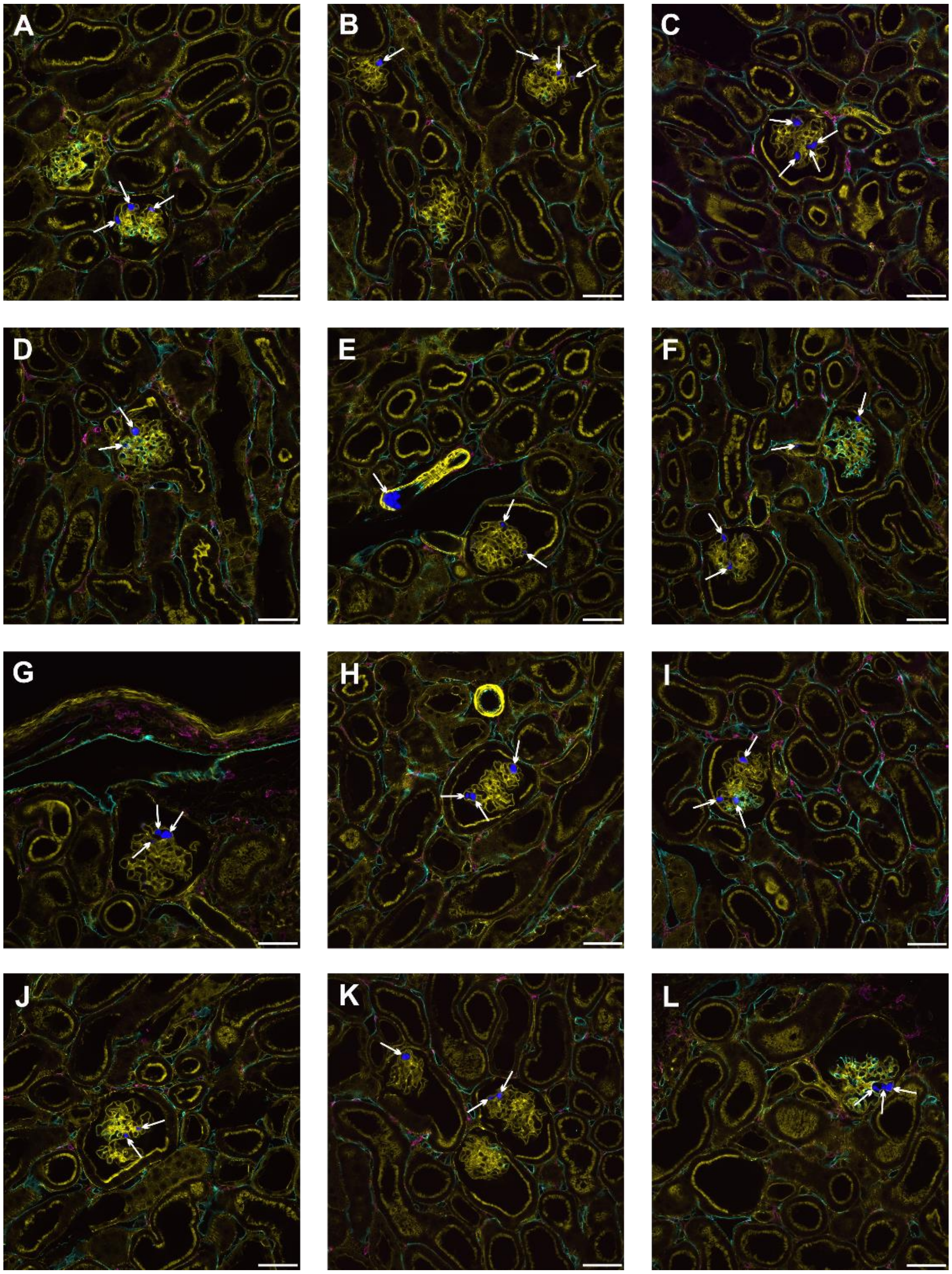

Figure S 15: Panel of twelve different areas in murine kidney tissue (A-L) (150 µm sections) of a MIPK, perfused with 0.5 mg DPA-PS MP (arrows; blue, 405 nm) after fluorescence immunohistochemistry staining for CD31 (endothelial cells; cyan, 647 nm), F4/80 (murine macrophages; magenta, 555 nm), and Phalloidin (f-actin; yellow, 488 nm). Scale bar: 50 µm.

REFERENCES

(1) Lakowicz, J. R. *Principles of fluorescence spectroscopy*, 3. ed.; Springer, 2006. DOI: 10.1007/978-0-387-46312-4.

(2) Taniguchi, M.; Lindsey, J. S. Database of Absorption and Fluorescence Spectra of 300 Common Compounds for use in PhotochemCAD. *Photochem. Photobiol.* **2018**, *94* (2), 290–327. DOI: 10.1111/php.12860.

(3) Schroter, A.; Märkl, S.; Weitzel, N.; Hirsch, T. Upconversion Nanocrystals with High Lanthanide Content: Luminescence Loss by Energy Migration versus Luminescence Enhancement by Increased NIR Absorption. *Adv. Funct. Mater.* **2022**, *32* (26), 2113065. DOI: 10.1002/adfm.202113065.

(4) Himmelstoß, S. F.; Hirsch, T. Long-Term Colloidal and Chemical Stability in Aqueous Media of $NaYF_4$ -Type Upconversion Nanoparticles Modified by Ligand-Exchange. *Part & Part Syst Charact* **2019**, *36* (10), 1900235. DOI: 10.1002/ppsc.201900235.

(5) Ajee, R. S.; Roy, P. S.; Dey, S.; Sundaresan, S. Upconversion nanoparticles and their potential in the realm of biomedical sciences and theranostics. *J Nanopart Res* **2024**, *26* (3), 1–17. DOI: 10.1007/s11051-024-05960-1.

(6) Dong, A.; Ye, X.; Chen, J.; Kang, Y.; Gordon, T.; Kikkawa, J. M.; Murray, C. B. A generalized ligand-exchange strategy enabling sequential surface functionalization of colloidal nanocrystals. *J. Am. Chem. Soc.* **2011**, *133* (4), 998–1006. DOI: 10.1021/ja108948z.

(7) Mahfoudh, A.; Barbeau, J.; Moisan, M.; Leduc, A.; Séguin, J. Biocidal action of ozone-treated polystyrene surfaces on vegetative and sporulated bacteria. *Appl. Surf. Sci.* **2010**, *256* (10), 3063–3072. DOI: 10.1016/j.apsusc.2009.11.074.

(8) Sarkar, A. K.; Rubin, A. E.; Zucker, I. Engineered Polystyrene-Based Microplastics of High Environmental Relevance. *Environ. Sci. Technol.* **2021**, *55* (15), 10491–10501. DOI: 10.1021/acs.est.1c02196.

(9) Eitzen, L.; Paul, S.; Braun, U.; Altmann, K.; Jekel, M.; Ruhl, A. S. The challenge in preparing particle suspensions for aquatic microplastic research. *Environ. Res.* **2019**, *168*, 490–495. DOI: 10.1016/j.envres.2018.09.008.

(10) Hrovat, B.; Uurasjärvi, E.; Viitala, M.; Del Pino, A. F.; Mänttäri, M.; Papamatthaiakis, N.; Haapala, A.; Peiponen, K.; Roussey, M.; Koistinen, A. Preparation of synthetic micro- and nano plastics for method validation studies. *Sci. Total Environ.* **2024**, *925*, 171821. DOI: 10.1016/j.scitotenv.2024.171821.

(11) Schmidt, J.; Plata, M.; Tröger, S.; Peukert, W. Production of polymer particles below 5 μm by wet grinding. *Powder Technol.* **2012**, *228*, 84–90. DOI: 10.1016/j.powtec.2012.04.064.

(12) Esch, E. von der; Lanzinger, M.; Kohles, A. J.; Schwaferts, C.; Weisser, J.; Hofmann, T.; Glas, K.; Elsner, M.; Ivleva, N. P. Simple Generation of Suspensible Secondary Microplastic Reference Particles via Ultrasound Treatment. *Front. Chem.* **2020**, *8*, 169. DOI: 10.3389/fchem.2020.00169.

(13) Choi, D.; Bang, J.; Kim, T.; Oh, Y.; Hwang, Y.; Hong, J. In vitro chemical and physical toxicities of polystyrene microfragments in human-derived cells. *J. Hazard. Mater.* **2020**, *400*, 123308. DOI: 10.1016/j.jhazmat.2020.123308.
